# Gram-negative synergy and mechanism of action of alkynyl bisbenzimidazoles

**DOI:** 10.1038/s41598-019-48898-4

**Published:** 2019-10-02

**Authors:** Jordan Chamberlin, Sandra Story, Nihar Ranjan, Geoffrey Chesser, Dev P. Arya

**Affiliations:** 1 Department of Chemistry, Clemson University, Clemson, 29631 USA; 2 NUBAD LLC, Greenville, 29605 USA; 3 Present Address: Department of Medicinal Chemistry, National Institute of Pharmaceutical Education and Research (NIPER) Raebareli, Lucknow, Uttar Pradesh, 226002 India

**Keywords:** Drug discovery and development, Drug discovery and development

## Abstract

Bisbenzimidazoles with terminal alkynyl linkers, selective inhibitors of bacterial topoisomerase I, have been evaluated using bacterial cytological profiling (BCP) to ascertain their mechanism of action and screened for synergism to improve Gram-negative bacterial coverage. Principal component analysis of high throughput fluorescence images suggests a dual-mechanism of action affecting DNA synthesis and cell membrane integrity. Fluorescence microscopy of bacteria challenged with two of the alkynyl-benzimidazoles revealed changes in the cellular ultrastructure that differed from topoisomerase II inhibitors including induction of spheroplasts and membrane lysis. The cytoskeleton recruitment enzyme inhibitor A22 in combination with one of the alkynyl-benzimidazoles was synergistic against *Acinetobacter baumannii* and *Escherichia coli*. Gram-positive coverage remained unchanged in the A22-alkynyl bisbenzimidazole combination. Efflux inhibitors were not synergistic, suggesting that the Gram-negative outer membrane was a significant barrier for alkynyl-bisbenzimidazole uptake. Time-kill assays demonstrated the A22-bisbenzimidazole combination had a similar growth inhibition curve to that of norfloxacin in *E.coli*. Bisbenzimidazoles with terminal alkynyl linkers likely impede bacterial growth by compromising cell membrane integrity and by interfering with DNA synthesis against Gram-positive pathogens and in the synergistic combination against Gram-negative pathogens including *E. coli* and multidrug-resistant *A. baumanii*.

## Introduction

There is a dire need for new classes of antibiotics with novel mechanisms of action (MOA), for which there are currently few options. Antibiotic resistance, especially among Gram-negative bacteria, is rising rapidly, and the World Health Organization considers the development of novel targets to counter these pathogens extremely important^1^. One such potential target is bacterial topoisomerase I, an enzyme critical for DNA replication^2,3^. Support for such enzymes as viable targets in need of further development is the extended use of fluoroquinolones that target bacterial topoisomerase II and IV which has resulted in the emergence of resistant bacteria. This precedence has encouraged further development of novel drugs that target topoisomerases^4^.

Bisbenzimidazoles used here are Hoechst 33258 inspired compounds, historically used as DNA binding dyes for their ability to specifically interact with adenine and thymine rich regions of the minor groove of DNA^5^. Additions of hydrophobic groups to bisbenzimidazoles have been shown to convert these non-specific bisbenzimidazoles to bacterial topoisomerase I specific inhibitors, as previously reported^6–9^. Bisbenzimidazoles with a propylpiperazine moiety and a para-ethoxy terminal group were demonstrated to have significant activity against *Escherichia coli* and specificity towards bacterial topoisomerase I with little effect on topoisomerase II or human topoisomerase I. This study supports that bisbenzimidazoles with hydrophobic moieties have potential for further target drug development^6^. More recently, these bisbenzimidazole derivatives were described to have broad spectrum bacterial coverage when used in combination with carbonyl cyanide 3-chlorophenylhydrazone, an efflux pump inhibitor^7^.

Recently, we have reported the synthesis and activity of several bisbenzimidazoles modified with the addition of terminal alkynyl linkers which effectively inhibited the growth of methicillin-resistant *Staphylococcus aureus* (MRSA) and expressed selectivity towards bacterial topoisomerase I^8,9^. Additionally the alkynyl-benzimidazoles were predicted to be less genotoxic as compared to the parent dye compounds, attributed to their significantly lower affinity for DNA duplexes when compared to Hoechst 33342 or Hoechst 33258, as measured by the thermal stabilization of DNA duplexes. These findings suggest that the DNA binding capabilities of bisbenzimidazoles with alkynyl linkers may be uncoupled from that of the topoisomerase I binding activity, thereby reducing mutagenicity and genotoxicity as compared to similar compounds and generated our present interest to further examine the MOA of these modified bisbenzimidazoles^8,9^.

So far, bisbenzimidazoles have not been analyzed for their MOA in bacterial cells. Biochemical studies suggest topoisomerase I inhibition but given the history of these drugs as DNA binding agents, further analysis is needed for confirmation. Macromolecular synthesis assays are used to determine the MOA for novel antibiotics; however, this method is slow, tedious, and therefore not applicable to high-throughput screening of multiple compounds^10^. Recently, bacterial cytological profiling (BCP) has been described as an alternative method for identifying the MOA of newly discovered compounds using live bacterial cultures and high-throughput screening of fluorescent images. BCP uses both qualitative fluorescence imaging and quantitative principal component analysis to group compounds according to their cytological impact and can be a powerful tool to rapidly assess the MOA of hundreds of compounds with a variety of bacteria^11^. BCP is applicable to many different species of bacteria in a variety of investigations that include rapid identification of antibiotic arsenals, novel mechanism of action, and phenotypic profiling of inhibition pathways^11–15^. BCP has been used to characterize the MOA of novel cell wall and RNA synthesis inhibitors across various strains of bacteria including MRSA, *Escherichia coli*, and *Bacillus subtilis*^14,15^. Cytological profiling is remarkably versatile with the capability to discriminate novel mechanisms outside the traditional targets as evidenced by the previous analysis of spirohexenolide A, a compound that collapses the proton-motive force^11^. Cytological profiling can also be applied to predict the observed morphology for a specific molecular target, dubbed rapid inhibition profiling, which further demonstrates the utility of fluorescence-microscopy methods for mechanistic analysis^12^.

**Figure 1:**
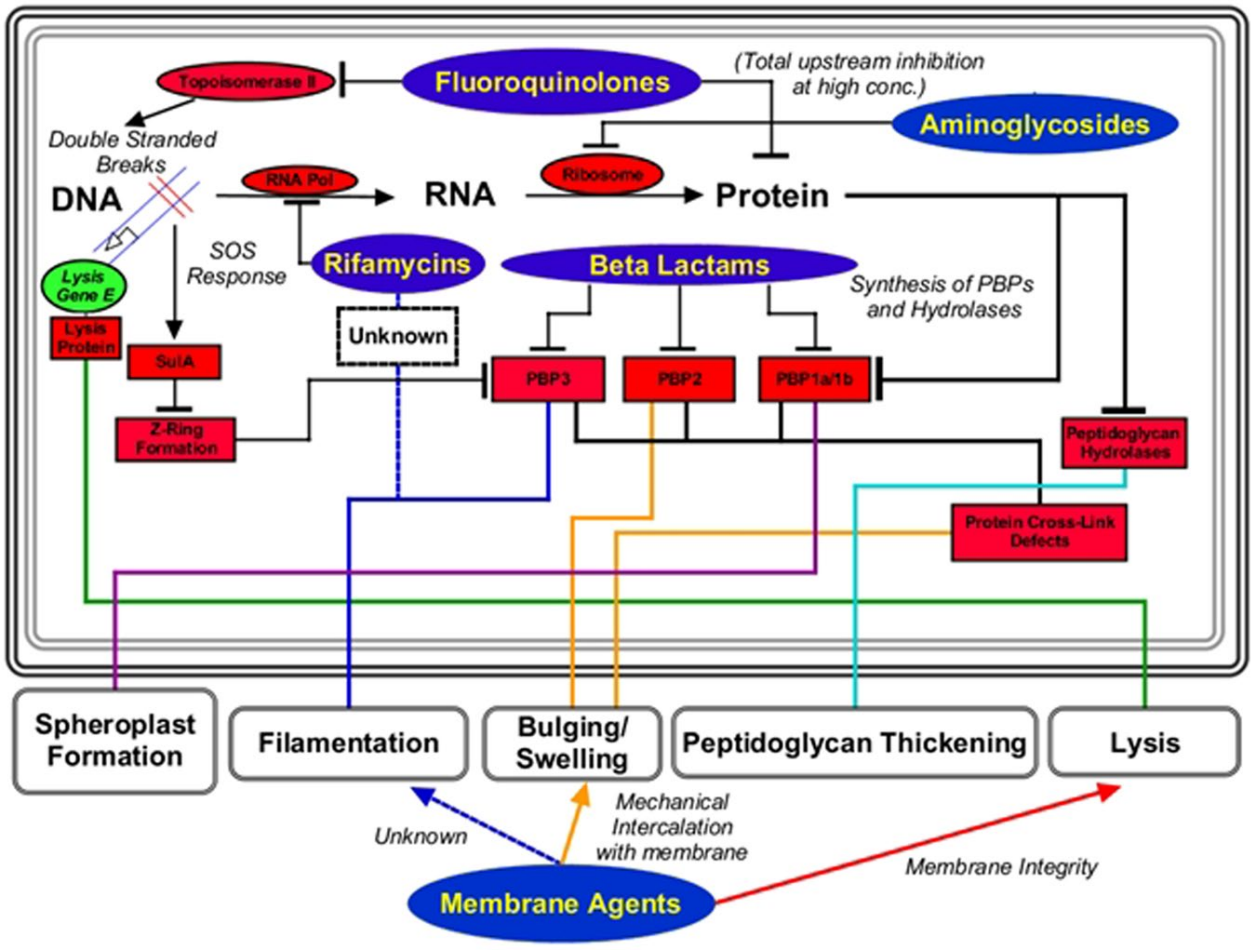
Summary of information in *Cushnie et al*.^16^. Many phenotypes are produced as either direct or indirect dysregulation of PBPs or induction of the SOS response. Cell-wall synthesis inhibitors act directly on the PBPs, but DNA/RNA/Protein synthesis inhibitors create upstream deficits which indirectly alter function of PBPs and result in cellular ultrastructure changes. Membrane-active agents also induce many of the same phenotypes but by different mechanisms such as mechanical intercalation or compromise of membrane activity. Understanding of differential change induced by antibiotics can be clearly correlated by cytological profiling and quantitative analysis.

The morphology of bacteria treated by classical antibiotics is well known using drugs that affect the 5 major biosynthetic pathways (DNA synthesis, RNA synthesis, cell wall synthesis, protein synthesis, and fatty acid synthesis). A recent review of the literature described the expected morphologies for the major inhibition targets of antibiotics used today and concluded that ultrastructure morphology for each group is reproducible and distinct^16^. Changes in the ultrastructural morphology due to drug exposure have many possible presentations (Fig. 1), but the main phenotypes seen are long filaments and rounded spheroplasts/ovoid cells with subtle variations in septal presence, septal width, cell wall shape, cell wall thickness, cross-link defects and perimortem observations such as lysate and ghost cells^16^. For example, specific dysregulation of penicillin-binding proteins that coordinate assembly of the peptidoglycan layer, the induction of the SOS response, or extracellular perturbations of the cell envelope induces major changes in cell morphology. Reproducibility and ease of observation allows for mechanistic predictions simply by measuring cell morphology parameters, a breakthrough in high-throughput screening.

While development of drugs that target multi drug-resistant (MDR) Gram-positive pathogens is a high priority, it is the MDR Gram-negative pathogens that are at the top of the World Health Organization’s (WHO) list that includes carbapenem-resistant *Acinetobacter baumanii*, carbapenem-resistant *Pseudomonas aeruginosa* and carbapenem-resistant extended-spectrum beta-lactamase producing *Enterobacteriaceae*^1^. We have previously demonstrated that alkynyl-bisbenzimidazoles are excellent inhibitors of Gram-positive bacteria but have a very limited Gram-negative coverage^8^ indicating the need for exploration of potential synergistic combinations and resolution of the chief barriers for Gram-negative efficacy – multi-drug efflux pumps and the outer membrane barrier^10^. In the present study we use BCP to further examine the mechanism of action of bisbenzimidazoles with terminal alkynyl linkers and apply synergistic drug combinations to improve Gram-negative coverage of these compounds to further advance the efficacy of these drugs.

## Results and Discussion

### Cytological profiling is a powerful tool for high-throughput screening of the mechanism of action of novel antibacterials

Bacterial cytological profiling (BCP) relies on the concept that antibacterial compounds with distinct mechanisms of action also produce distinct phenotypes in drug-exposed bacteria. A total of three different dyes (DAPI, a DNA intercalating agent, FM4-64, a cell membrane staining dye, and SYTOX-Green, a dye impermeable to intact cells) were used in accordance to the method devised in *Nonejuie et al*.^11^. Different classes of antibiotics induce different phenotypes in bacteria through the dysregulation of a handful of proteins or pathways (Fig. 1). Most of the measurable ultrastructural changes occurs through the inhibition of penicillin binding proteins (PBPs) that catalyze the formation of the peptidoglycan layer, and have long been known to affect morphology and cellular shape in bacteria. They also serve as a critical pivot for observing cell morphology using electron microscopy and cytological profiling^16–18^. β-lactams directly inhibit PBPs by inducing cell filamentation, bulging, swelling, peptidoglycan thickening, spheroplast formation, and cross-link defects. Aminoglycosides inhibit protein synthesis, and ultimately dysregulate the assembly of peptidoglycan through mistranslation. Aminoglycosides invoke a spheroplastic phenotype by the downstream inhibition of proper PBP function, a conclusion substantiated by the evidence from mutant studies^16,19^. Fluoroquinolones induce the SOS pathway by induction of double-stranded DNA breaks from the ternary complex formed with DNA, topoisomerase II, and fluoroquinolone, resulting in bacteriostasis and ultimate cell death through oxidative stress^20^. However, since many different classes of antibiotics converge on similar observations, the combination of each cellular parameter must be examined individually to determine the likely mechanism of action in any new drug.

**Figure 2:**
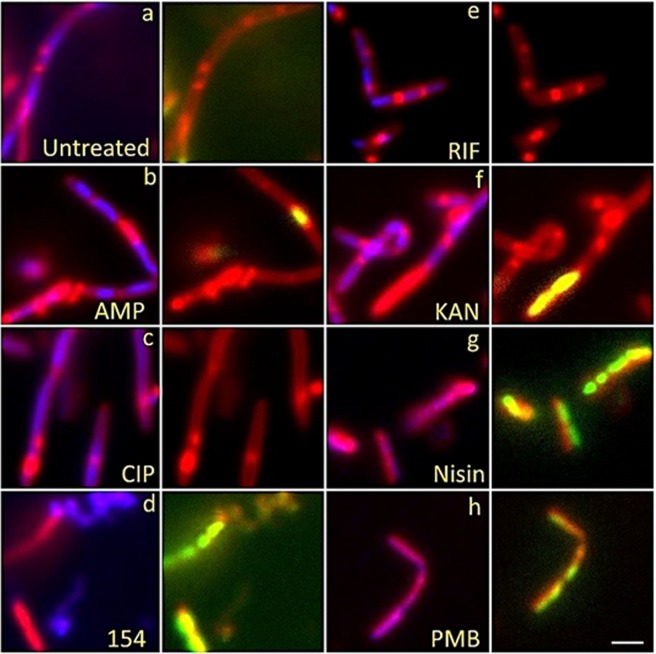
Fluorescent microscopy images of *B. subtilis* 6051 treated with DAPI (Blue), SytoxG (Green) and Fm4-64 (Red) treated with traditional antibiotics influencing each biosynthetic class (DNA synthesis, RNA synthesis, cell wall synthesis, protein synthesis, and membrane-active) and test compound DPA 154, suspected of being a DNA synthesis inhibitor by way of topoisomerase 1A inhibition. DPA 154 produces lysed cell contents similar to membrane-active compounds and DNA morphology similar to ciprofloxacin. Ampicillin produces filamentous cells with membrane blebbing and membrane permeation. Ciprofloxacin produces filamentous cells with reduced DNA content as seen by DAPI and a lack of permeated membranes. Rifampicin produces moderate cell lengthening and unilateral membrane bulging seen as dense membrane enhancement by FM4-64. Kanamycin procures peptidoglycan thickening, bulging, and moderate filamentation. Nisin and polymyxin B both cause cell lysis and production of ovoid cells due to compromised cell membrane. Bacteria shown in paired images; DAPI + FM4-64 (left) and FM4-64 + SytoxG (right). **(a)** Untreated cells. Treatment with **(b)** ampicillin at five times MIC (1 µg/mL), **(c)** ciprofloxacin at five times MIC (0.025 µg/mL), **(d)** 154 at 10 µg/mL, **(e)** rifampicin at five times MIC (0.025 µg/mL), **(f)** kanamycin at five times MIC (10 µg/mL), **(g)** Nisin at five times MIC (10 µg/mL), and **(h)** polymyxin B at five times MIC (5 µg/mL). Further description of expected phenotypes may be found in Supplementary Data (Tables S1–S6). Large composite images of each antibiotic may also be viewed in Supplementary Data (Figs S5–S18). Scale bar 1 µM.

Cytological profiling of *Bacillus subtilis* 6051 stained with DAPI (Blue), SYTOX GREEN (Green) and FM 4-64 (Red) treated with known antibiotics from each major biosynthetic class and test compound DPA 154 is given in Fig. 2. The DNA synthesis inhibitor ciprofloxacin demonstrates major filamentous growth, the protein synthesis inhibitor kanamycin demonstrates minor filamentation, spheroplastic phenotypes, and peptidoglycan thickening, while the cell wall inhibitor ampicillin causes bulging, filamentation and loss of membrane integrity. The RNA synthesis inhibitor rifampicin induces minor filamentation septal wall disruptions, and membrane-active compounds nisin and polymyxin-B cause membrane damage demonstrated by uptake of SYTOX GREEN. DPA 154 also causes membrane damage as seen by the uptake of SYTOX GREEN as well as induction of spheroplastic phenotypes, suggesting a second membrane-active mechanism of action. It is important to note that while untreated *B. subtilis* cells are typically rods growing in filamentous chains which may be difficult to distinguish from elongated “filamentous” phenotypes, differentiation is possible from analysis of individual cell boundaries where antibiotic treated cells will have longer, less segmented chains representing fewer individual bacterial cells per field. Further description of the expected phenotypes for each antibiotic class can be found in the Supplementary Materials (Tables S1–S6).

### Evaluation of alkynyl-bisbenzimidazoles by BCP

**Figure 3:**
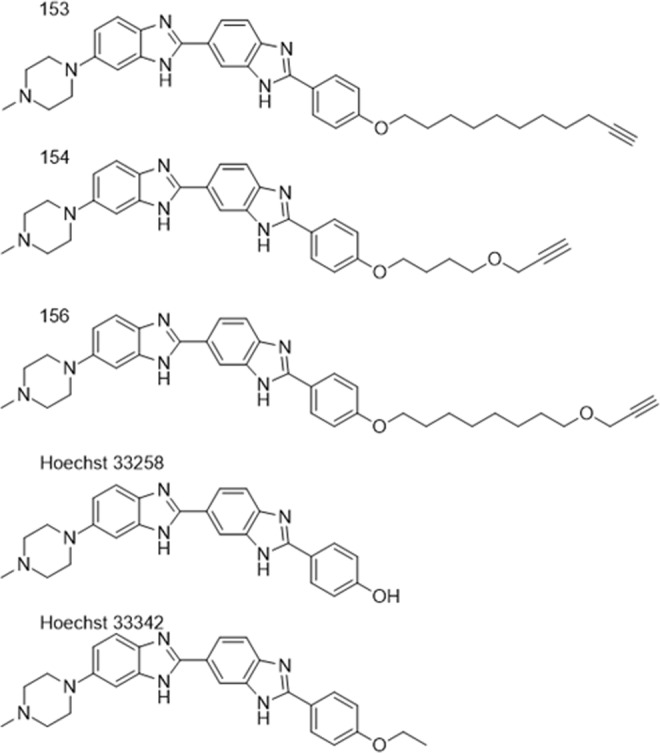
Bisbenzimidazoles used in this study. Addition of terminal alkynyl linkers to Hoechst 33258-derived bisbenzimidazoles has been previously shown to confer specificity for bacterial topoisomerase I over human topoisomerase or topoisomerase II. DPA 153 (bacterial studies), DPA 154 (bacterial studies and cytological profiling), DPA 156 (bacterial studies and cytological profiling), and Hoechst 33258/33342 (bacterial studies).

Bisbenzimidazoles have been shown to inhibit bacterial topoisomerase IA independently many times across a wide variety of compounds sharing the bisbenzimidazole backbone, leading to expectations of a possible novel antibacterial mechanism^7–9^. Docking studies have proposed that bisbenzimidazoles bind to the active site of topoisomerase IA, forming a ternary complex with DNA and the enzyme, and inducing double-stranded DNA breaks which can lead to cell death. We hypothesized that the addition of terminal alkynyl linkers to benzimidazoles (Fig. 3) would alter or add to the MOA of the benzimidazole interacting with topoisomerase I. Previously our docking studies indicate that the terminal alkynyl linker makes critical interactions with bacterial topoisomerase I, shifting the molecule in the active site, and imparting specificity for the bacterial enzyme homologue^8^. Thus, we expect similar interactions as the fluoroquinolones, which inhibit bacterial topoisomerase II^21^.

**Figure 4:**
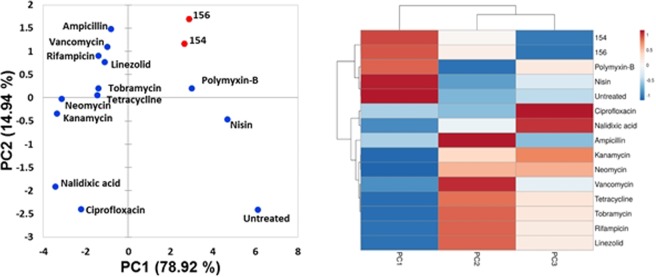
2D Principal component analysis of antibiotic-treated *B. subtilis* 6051 using unweighted variables from cytological imaging with associated agglomerative hierarchical clustering and heat map made in ClustVis^47^. PCA demonstrates DPA 154 and 156 induce morphology resembling membrane-active compounds polymyxin-B and nisin. Three independent wells of bacteria were treated with each antibiotic and 4 fields/well were imaged and the corresponding segmentation metrics were averaged. DPA compounds (154 and 156) appear to be more closely related to membrane active compounds polymyxin-B and nisin than DNA synthesis inhibitors such as ciprofloxacin and nalidixic acid. Principal components (PC) are the variable reduction outputs of the PCA algorithm that contribute a percentage of the variation of a sample; PC1 (78.92%) vs PC2 (14.94%). Variables that contribute to each principal component/factor are summarized in the supplemental figures (Table S8, Figs S3 and S4, Tables S12 and S13). A list of antibiotics and mechanisms of action used for cytological profiling may be found in Table S6. Definitions of select measures used in cytological profiling may be found in Table S9.

Principle component analysis (PCA) of *B. subtilis* demonstrates that DPA 154 and 156 behave more like membrane active compounds such as polymyxins than DNA synthesis inhibitors such as ciprofloxacin (Fig. 4). While polymyxin B is typically used to treat Gram-negative bacteria, wild-type strains of *B. subtilis* are susceptible to polymyxins, further supporting the membrane-active effect of DPA 154^22^.

**Figure 5:**
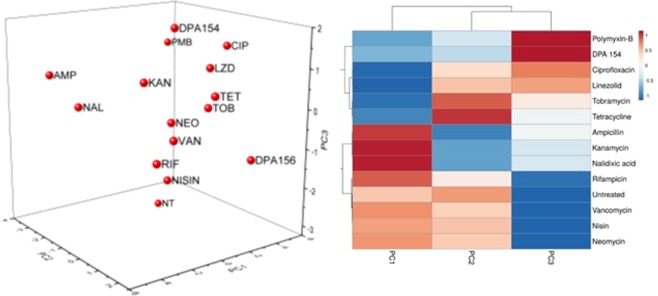
3D Principal component analysis of antibiotic-treated *E. coli* 25922 using unweighted variables from cytological imaging with associated agglomerative hierarchical clustering (AHC) and heat map made in ClustVis^47^. PCA demonstrates ambiguous association with any antibiotic class but AHC demonstrates close association with both membrane active compounds and DNA synthesis inhibitors. Three independent cultures of bacteria were treated with each antibiotic and the corresponding segmentation metrics were averaged. PC1 (51.95%) vs PC2 (18.11%) vs PC3 (11.87%). 3D scatterplot was used for *E. coli* and not *B. subtilis* because only two principal components insufficiently describes variation in *E. coli* morphology based on cumulative variability. Two components were sufficient to capture >90% of cumulative variability in *B. subtilis*. DPA 154 appears to be more closely related to the action of polymyxin-B than ciprofloxacin, but clear similarity between both is demonstrated in the clustering heat map. Summary of variables that contribute to each principal component/factor, parameters for cytological profiling, and bacterial measurements may be found in the supplemental figures (Figs S1 and S2, Tables S7, S12 and 13).

DPA 154 similarly impacted *E. coli* and was grouped with polymyxin B (Fig. 5). The PCA data alone does not indicate DPA 154 as most similar to membrane-active compounds because only 51.95% variation is attributed to the first principal component and, therefore, the 3^rd^ principal component is not shown. However, when the first three principal components are analyzed by hierarchical clustering, DPA 154 appears most similar to polymyxin B.

DPA 154 has a shorter alkynyl chain (as compared to DPA 156) and showed DNA morphology similar to ciprofloxacin in *B. subtilis* providing evidence that DPA 154 is a dual-mechanism inhibitor. DPA 156 has a longer alkynyl linker (10 atoms) than DPA 154 (6 atoms), but has almost no antibacterial activity despite greater hydrophobicity. This finding suggests the DPA 154 makes critical interactions with the membrane and potentiates the antibacterial activity through an unknown target. The *E. coli* strain used in this study was a wild-type strain (ATCC 25922), as opposed to previous studies which used a membrane-permeable mutant *E. coli LptD4213*^11^. Thus, the failure for DPA 156 to demonstrate results similar to DPA 154 is likely due to the outer membrane barrier, this observation supports our previous work that demonstrated DPA 156 to have less activity in Gram-negative bacteria^8^.

### Fluorescence microscopy and quantitative analyses provide evidence for DPA 154 as dual mechanism inhibitor

**Figure 6:**
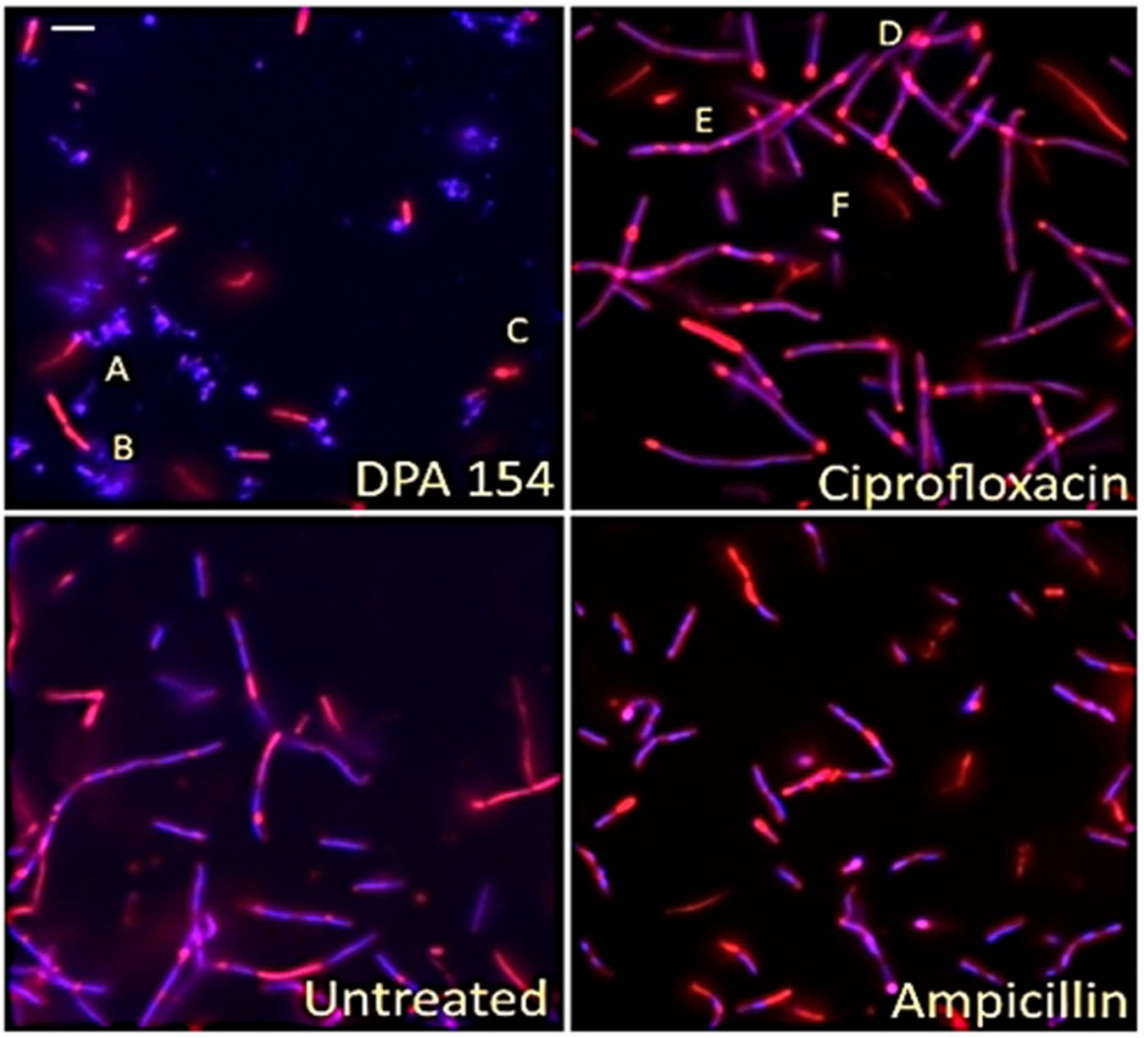
Fluorescence microscopy images of DPA 154, ciprofloxacin, ampicillin, and untreated *B. subtilis* 6051 at five times MIC of each compound after addition of DAPI (blue) and FM 4-64 (Red) for 2 hours demonstrating clear difference in mechanism of action. DPA 154 induces lysed cells and ovoid cells consistent with membrane-permeating compounds. Ciprofloxacin induces substantial cell lengthening and DNA morphology changes such as reduction in nucleoid count. Ampicillin induces membrane bulging and moderate cell lengthening. (**A**) Cellular lysate seen as DAPI stained nucleic acids. (**B**) Filamentous remnant. **(C)** Spheroplastic cell. (**D**) Bulging of cytoplasmic membrane. (**E**) Filamentous phenotypes. (**F**) Spheroplastic cell. Additional cytological profiles of *B. subtilis* may be viewed in the Supplemental Data (Figs S5–S18). Expected cytological profile for fluoroquinolones may be seen in the Supplemental Data (Table S2). Scale bar 1 µm.

**Figure 7:**
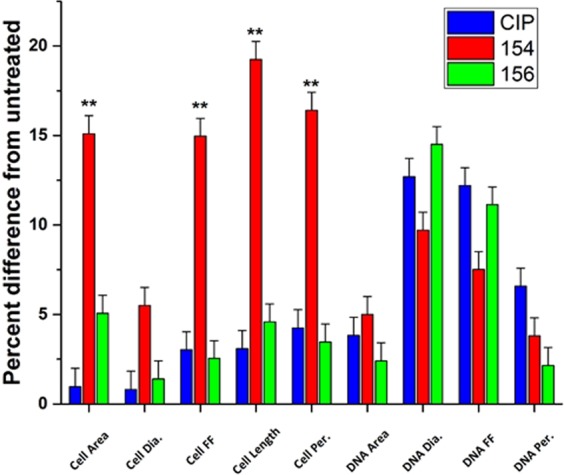
Comparison of DPA 154, 156, and ciprofloxacin-treated *E. coli* 25922 by percent difference from untreated across cellular and DNA parameters including area, diameter, length form factor (FF) and perimeter. DPA 154 and 156 produce significant changes in cell area, form factor, length, and perimeter, but statistically insignificant changes in DNA morphology. DPA 154 and 156 exert changes in DNA morphology like ciprofloxacin, but DPA 154 also induces changes in cellular ultrastructure inconsistent with DNA synthesis inhibition (cell area, diameter, form factor, length, perimeter), providing evidence for a dual mechanism of action. Statistical analysis of variances with post-hoc test can be found in the Supplementary Data (Table S15). *p < *0.05*; **p < *0.01*. Error bars represent 95% confidence interval for true value of the mean.

Fluorescence microscopy imaging demonstrates two distinct mechanisms of action induced by DPA 154. First, there is a clear difference in the phenotypes exerted by DPA 154 and ciprofloxacin, an inhibitor of DNA gyrase, on *B. subtilis* (Fig. 6). Ciprofloxacin-induced changes included filamentation and reduction in nucleoid count which were also seen in DPA 154 treated cells (Fig. 7). However, cell lengthening may also be caused by other classes of antibiotics such as aminoglycosides and β-lactams, so identification of additional cell morphologies is critical. DPA 154 also caused significant lysis of bacterial cells (Fig. 6), a phenotype not typically seen with topoisomerase II inhibitors^16^. Though there were filamentous remnants, DPA 154 also induced smaller, rounded spheroplastic cells. Spheroplasts are osmotically sensitive and are atypical of DNA synthesis inhibitors except at high concentrations, which is indicative of the assay condition with exposure of cells to five times its MIC value^23^. DNA synthesis inhibition was therefore evident for both DPA 154 and ciprofloxacin by the retention of DNA morphology, but a membrane-active mechanism was only observed with DPA 154 as supported by the significant changes in membrane morphology and appearance of cellular lysate, indicating two distinct mechanisms of action (Figs 6 and 7).

**Figure 8:**
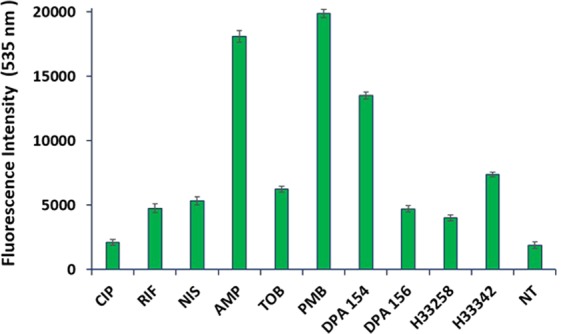
Comparative analyses of the proportion of SYTOX Green permeated *E. coli* cells treated with DPA 154, DPA 156 and antibiotics at five times MIC for 3 h. DPA 154 induced a much greater SYTOX Green uptake than ciprofloxacin and DPA 156, indicating a breakdown in membrane competence more consistent with membrane-active compounds. Aminoglycosides, RNA synthesis inhibitors, and fluoroquinolones induce much less membrane compromised cells than cell membrane or cell wall active compounds. Abbreviations: CIP ciprofloxacin, NIS nisin, RIF rifampicin, AMP ampicillin, TOB tobramycin, PMB polymyxin-B, H33258, H3342 Hoechst dye controls, NT no treatment. Error bars represent the standard deviation from the mean for assays performed on three occasions in duplicate. Culturable numbers of *E. coli* determined after each treatment may be viewed in the Supplementary Data (Fig. S24). MICs for antibiotics used may also be found in Table S18.

**Figure 9:**
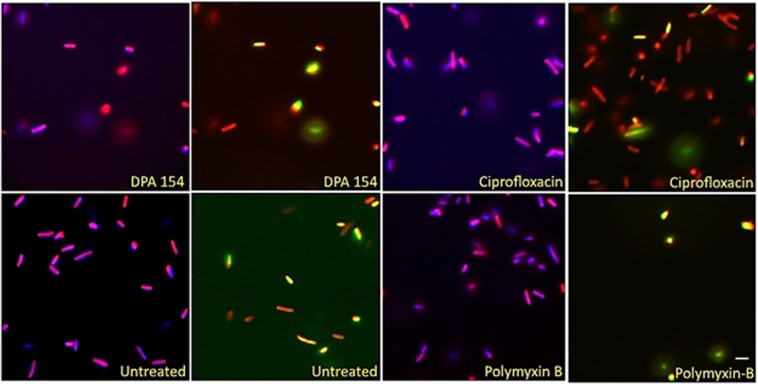
Comparison of *E. coli* treated with DPA 154, ciprofloxacin, and polymyxin-B. DPA 154 causes induction of spheroplastic phenotype like polymyxin-B and diminished nuclear morphology consistent with ciprofloxacin. Presence of features of both morphologies provides evidence for a dual mechanism of action for DPA 154. *E. coli* stained with DAPI (blue) FM4-64 (red) and SytoxG (green) and treated with DPA 154, ciprofloxacin, and polymyxin-B demonstrating membrane permeating effects of DPA 154. DPA 154 induces a high rate of smaller, rounded, SytoxG permeated cells consistent with membrane permeating antibiotics. Ciprofloxacin induces a longer, filamentous phenotype and cells with fewer membrane disruptions as compared to polymyxin-B which induced a majority of small, rounded, membrane permeated cells. Scale bar 1 µm.

Our previous work indicated that alkynyl benzimidazoles could inhibit DNA synthesis by interfering with bacterial topoisomerase I and these findings are corroborated with the evidence from quantitative analyses^8^. Fluoroquinolones alter DNA morphology by creating ternary complexes with DNA, topoisomerase, and fluoroquinolone, ultimately irreversibly altering DNA gyrase or topoisomerase IV form and function, resulting in double-stranded DNA breaks and halting DNA replication^4^. Ultrastructurally, DNA synthesis inhibitors exhibit filamentation in a time-dependent manner by activating the SOS response before ultimately collapsing into cells with similar size to the untreated cohort^23^. The alkynyl benzimidazoles similarly reduce the nucleoid count, but fail to induce the same filamentation and morphology changes as the fluoroquinolones (Fig. 7). DPA 154 is the only compound to induce both cellular ultrastructure changes and DNA morphology changes, suggesting a dual mechanism of action involving DNA replication enzymes and altered membrane integrity. DPA 154 and 156 both exert similar changes in DNA morphology in *E. coli*, indicating that the length of the alkynyl linker is important for membrane effects in Gram-negatives (Fig. 7). Additionally, DPA 154-treated *E. coli* cells accumulated a much higher intracellular SYTOX GREEN concentration (Figs 8 and 9) but failed to permeabilize the membrane, a conclusion corroborated by moderate growth inhibition relative to the control Hoechst dye demonstrated in prior studies^8^.

### Synergistic interactions of antibiotics in combination with alkynyl benzimidazoles

Previously we demonstrated that alkynyl benzimidazoles have broad Gram-positive coverage and limited Gram-negative coverage. The Gram-positive bacteria MRSA, methicillin susceptible *S. aureus* (MSSA), *Staphylococcus epidermis*, and *Enterococcus faecium* were susceptible with relatively low MIC values (1–4 µg/mL), indicating these compounds may have potential for therapeutic use. Gram-negative coverage could be improved as these compounds only moderately inhibits Gram-negative pathogens such as *Acinetobacter baumannii*, *Pseudomonas aeruginosa*, and *E coli* with MIC values of 8–16 µg/mL and some Gram-negative bacteria appeared to be unaffected, such as capsule-producing *Klebsiella pneumoniae* and *Enterobacter cloacae* (MIC > 32 µg/mL)^8^.

To improve the bacterial susceptibility to alkynyl benzimidazoles we tested different antibiotic combinations with a variety of ***ESKAPE*** pathogens (*Enterobacter*, *Staphylococcus*, *Klebsiella*, *Acinetobacter*, *Pseudomonas and Enterococcus*) that commonly have multiple intrinsic and acquired drug resistance mechanisms. Combination therapy for Gram-negative infections is commonly used in clinical settings in treatment of sepsis as early combination therapy is associated with improved survival and decreased antibiotic resistance development^24^. Additionally, the rise of antibiotic resistance posits the need for drugs with broad spectrum activity, so formulations that improve the spectrum of activity are inherently valuable outcomes for drug development^25^. The bacteria chosen for synergy assays were selected for their low susceptibility in prior studies, notably *A. baumannii*, *K. pneumoniae*, and *P. aeruginosa*. Previously we found that the control Hoechst compounds (33258 and 33342) vary in their activity against different strains and acutely inhibit growth of a variety of pathogens, but both are known mutagens. However, DPA compounds 153, 154 and 156 show preference for bacterial topoisomerase IA only, and as such are more selective^8,26^. A variety of compounds were assayed for synergy in combination with DPA 153, 154 and 156 that included membrane active compounds and efflux pump inhibitors (Fig. S19, Tables S16 & S17)^27^. Previous studies of other bisbenzimidazole libraries posit bisbenzimidazoles as substrates of active efflux, indicating combination with efflux inhibitors may be an effective route for improvement of coverage^7^. Alkynyl-bisbenzimidazole compounds contain long hydrophobic alkynyl linkers that may interact with the outer membrane of Gram-negative bacteria, therefore combination efficacy with membrane active antibiotics was investigated. In a high throughput single point synergy screen four compounds showed potential in coordination with alkynyl bisbenzimidazoles: phenylalanine-arginine-β-naphthylamide (PAβN), a broad-spectrum efflux pump inhibitor, polymyxin-B and the nonapeptide variant which lacks the hydrophobic tail, and A22, a cytoskeletal recruitment enzyme inhibitor (Tables 1 and 2). The best combination in the single point synergy screening assay was with DPA 154 and A22, demonstrating the highest levels of growth inhibition (<1 µM DPA 154 for *K. pneumoniae* NR15410, *P. aeruginosa* 27853 and polymyxin B-resistant *E. cloacae*). PAβN has been demonstrated to resensitize rifaximin-resistant *E. coli* at similar concentrations, possibly explaining the remarkable drop (4–5 fold) in fractional inhibitory concentration for DPA 154 treated *E. coli* in combination with PAβN^28^. PAβN increases susceptibility to other drugs by inhibition of the resistance/nodulation/division efflux superfamily and has secondary membrane-acting effect possibly owing to its behavior as a cationic peptide^29^. This is a critical observation as the rest of the efflux pump inhibitors failed to sensitize *K. pneumoniae* and *P. aeruginosa* to DPA compounds, perhaps indicating that efflux was not the limiting characteristic for the activity of DPA 154 in these strains.

**Table 1:** Single-point synergy assay (SPSA) of DPA 154 in combination with membrane-active compounds against ***ESKAPE*** pathogens.

Bacteria	FIC DPA 154 (µM)				
	154	154 + PMB	154 + PMBN	154 + PAβN	154 + A22
*E. faecium* BM4105-RF	4	8	8	8	8
MRSA 33591	2–4	8	8	>32	4
*K. pneumoniae* NR15410	>32	8	16	32	>32
*A. baumannii* 19606	>32	>32	32	>32	16
*P. aeruginosa* 27853	>32	16	32	16	0.25
*E. cloacae* 13047	>32	>32	>32	>32	0.5
*E. coli* 25922	>32	16	16	2	0.25

In the SPS assay DPA 154 was diluted from 32–0.25 µM and the concentration of the membrane active compounds remained constant with PMB 0.39 µM, PMBN 100 µM, PAβN 100 µM and A22 32 µM. DPA 154 and A22 demonstrates marked reduction in DPA 154 necessary for inhibition of *ESKAPE* pathogens at intermediate concentrations. DPA 154 + PMB produces less robust results against Gram-positives than DPA 154 alone. DPA 154 and PMBN, a non-inhibitory PMB derivative, demonstrate similar results to DPA 154 and PMB. DPA 154 + PA PAβN produces strain specific results. Abbreviations: FIC fractional inhibitory concentration, PMB polymyxin-B, PMBN polymyxin-B nonapeptide, PAβN phenylalanine-arginine-β-naphthylamide, A22 S-(3,4-Dichlorobenzyl)isothiourea. See Supplemental Data (Table S16 and Fig. S19) for minimum inhibitory concentrations and structures of synergy compounds. See Table S16 for full single-point synergy assay results.

The polymyxins are well characterized membrane active compounds by way of binding to the lipid A portion of the lipopolysaccharide layer and destroying the outer membrane. Polymyxin B at a sub-inhibitory concentration of 0.4 µM was expected to be synergistic by compromising the outer membrane allowing increased concentrations of the test compound to reach the intracellular compartment^30^. Treatment of bacteria with the polymyxin-B nonapeptide (PMBN) – DPA 154 combination mirrored those of polymyxin-B with 2–3-fold reduction in MIC (Table S17). PMBN is non-toxic to bacterial cells, but still induces membrane permeability by binding to lipid A, interrupting the bridging divalent cations, and creating gaps between the head-groups^31^. Therefore, the observed inhibition strongly implies that outer-membrane permeability is a chief obstacle for inhibition of Gram-negative bacteria by DPA 154.

### The cytoskeletal inhibitor A22 potentiates DPA 154 in gram negative bacteria

**Figure 10:**
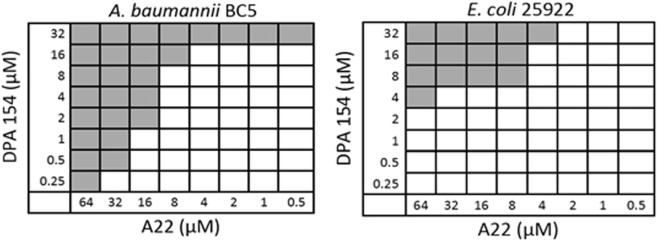
Examples of checkerboard synergy assay for the Gram-negative bacteria MDR *A. baumannii* BC5 and *E. coli* 25922 demonstrating synergy *E. coli* 25922 FICI = 0.10; A22 FICI = 0.006. Additional checkerboard plates are viewable in the Supplemental Material (Fig. S20).

**Figure 11:**
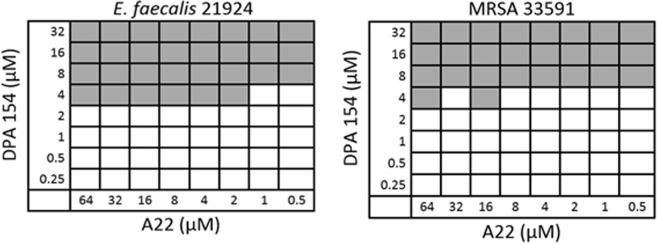
Examples of checkerboard synergy assay for Gram-positive pathogens (MRSA 33591 and *E. faecalis* 21924) demonstrating lack of synergy. MRSA 33591 FICI = 1.00; *E. faecalis* 29124 FICI = 0.25. Additional checkerboard assay plates are viewable in Supplementary Material (Fig. S20).

A22 inhibits proper formation of rod morphology and inhibits chromosome partitioning in rod-shaped bacteria and was the best synergistic compound overall in combination with DPA 154 against multi-drug resistant (MDR) *A. baumannii P. aeruginosa*, *K. pneumoniae*, and *E. coli* in our single point screen with an 8-fold reduction in the fractional inhibitory concentration (Table S17). Because the single point screen is vulnerable to false positives, checkerboard synergy assays were performed to verify these results (Table 2). Notably, MDR *E. coli* H4H and *A. baumannii* BC5 strains were most susceptible to the combination with FIC indices of 0.16 and 0.31 respectively with a significant reduction in the FIC of DPA 154 but not that of A22. Whereas with the Gram-positive cocci (*E. faecium*, *E. faecalis* and *Staphylococcus* spp.) no synergy was observed (Figs 10 and 11).

**Table 2:** Bacterial activity of DPA 154 in combination with A22 against ***ESKAPE*** pathogens.

Strain	MIC_a_	MIC_b_	FIC_a_	FIC_b_	FICI
*E. faecium* BM4105	4	>64	8	8	<2.13
*E. faecalis* 29121	16	>64	4	2	<**0.28**
*S. aureus* 25923	4–8	>64	8	1	<1.35
MRSA 33591	2–4	>64	4	16	<1.58
*K. pneumoniae* NR15410	>32	>64	16	16	<0.75
*K. pneumoniae* 1332	>32	>64	—	—	NS
*A. baumannii* BC5	>32	>64	2	16	<**0.31**
*A. baumannii* 19606	>32	>64	16	0.5	<0.51
*P. aeruginosa* 27853	>32	>64	0.25	64	<0.64
*E. cloacae* 13047	>32	6.25	0.25	4	<0.64
*E. coli* 25922	>32	>64	1	8	<**0.16**
*E. coli* H4H	32	>64	8	8	<**0.38**

Fractional inhibitory concentration indices (FICI) were determined using checkerboard microbroth dilution assay with maximum concentrations of DPA 154 and A22 of 32 µM and 64 µM, respectively. DPA 154 and A22 show synergism for select strains including multi-drug resistant *A. baumannii* and *E. coli*. A general lack of synergy for Gram-positive organisms is shown. MIC_a_ is the MIC of DPA 154 alone. MIC_b_ is the MIC of A22 alone. FIC_a_ is the FIC of DPA 154. FIC_b_ is the FIC of A22. FIC is calculated as the lowest inhibitory concentration in the presence of the synergy drug. FICI < 0.5 is considered synergistic; 0.5 < FICI < 4.0 is no interaction, and FICI > 4.0 antagonistic^48^. Synergy was observed in Gram-negative pathogens *K. pneumoniae* NR15410, *A. baumannii* BC5, *A. baumannii* 19606, *E. coli* 25922, and *E. coli* H4H.

**Figure 12:**
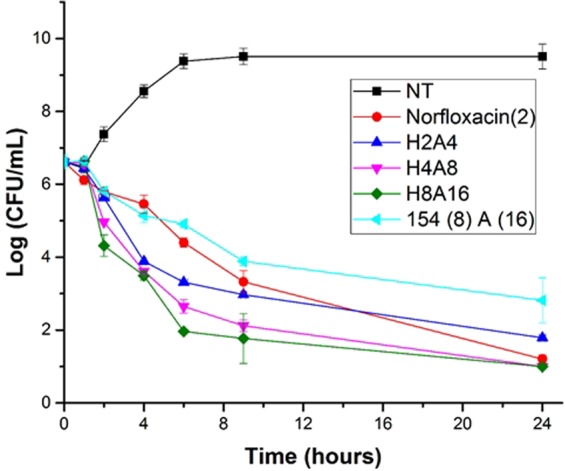
Time-kill assay evaluating *E. coli* 25922 treated with norfloxacin, combinations of Hoescht 33342 and A22, and DPA 154 in combination with A22. Legend: NT no treatment; Norfloxacin (2) Two times MIC (6.25 µM); 2 µM 33342 + 4 µM A22; 4 µM 33342 + 8 µM A22; 8 µM 33342 + 16 µM A22; 8 µM DPA 154 and 16 µM A22 (FIC = 0.1) The DPA 154 and A22 combination shows a similar time-kill kinetic curve to norfloxacin. Test agents were incubated with *E. coli* over a 24-hour period at 37 °C and bacteria culture was plated at 0, 1, 2, 4, 6, 8, and 24 hours for CFU/mL determination. Initial cell concentration was ~10^6^ cells/ml. Time-kill assay demonstrates DPA 154 and A22 is as effective as norfloxacin alone and quickly reduces viable cell count by 3 log-fold changes in 6 hours. Error bars represent standard deviation of CFU/mL of three replicates.

Time-kill assay using *E. coli* further verified the synergistic action of A22 and DPA154. Based on results from the checkerboard assays, the concentration of each compound combination was adjusted accordingly for the time kill assay. DPA 154 and A22 together inhibit bacterial growth at a rate most closely matched by norfloxacin alone (Fig. 12). This result suggests that DPA 154 and A22 together have the potential to be acute inhibitors with further modification of the bisbenzimidazole alkynyl group. A22 also showed remarkable synergy with Hoechst 33342 that was dose-dependent, suggesting A22 has broad synergy with compounds that target DNA synthesis. By 24 hours, DPA154 and A22 had reduced the number of colony forming units by a factor of 1000 with the greatest effect occurring in the first 8 hours. The Hoechst 33342-A22 combination was ultimately more effective than the DPA 154-A22 combination; however, because DPA 154 has greater selectivity for bacterial topoisomerase I, is less genotoxic, and less toxic to mammalian cells it represents a more viable combination for further development^8^.

**Figure 13:**
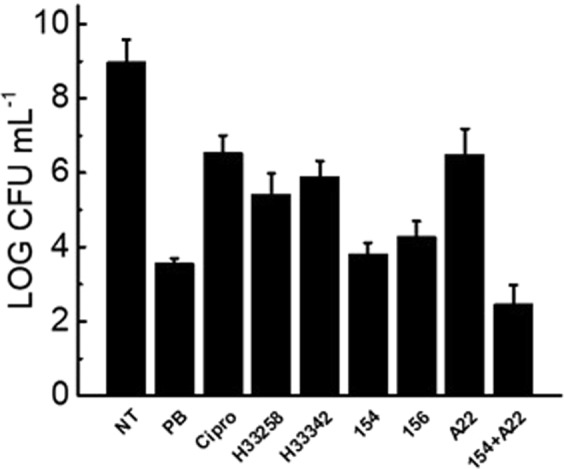
Culturable enumeration at BCP conditions (2 hours at five times MIC) demonstrating antimicrobial activity of compounds by a second method. All compounds exhibit >2 log-fold reduction in culturable units, providing supporting evidence for observed BCP phenotypes to be indicative of significant antimicrobial action. DPA 154 and 156 significantly decrease viable cell count at 2 hours similar to polymyxin-B. DPA 154/A22 combination reduced culturable cell count greater than either DPA 154 or A22 alone, supporting synergistic interaction demonstrated in synergy studies. Hoechst dyes induced less reduction in viable cells at 2 hours than DPA 154/156 test compounds. NT (No treatment), Cipro (Ciprofloxacin), H (Hoechst dye control), PB (Polymyxin-B), A22 (S-(3,4-dichlorobenzyl)isothiourea. Mid exponential phase cultures of *E. coli* were used for antibiotic challenge. Error bars represent two individual experiment repeats with two duplicates.

Validation of this mode of action was observed when we examined cell viability and membrane integrity when bacteria were challenged with the bisbenzimidizoles alone or in combination with A22 against *E. coli* under BCP conditions as shown in Fig. 13. DPA 154 and 156 both exhibited a 5-fold reduction in log CFUs/mL at five times MIC at 2 hours, while DPA 154/A22 combination exhibits a 6–7 fold reduction in log CFUs/mL at the same breakpoint. A22 itself reduced the colony forming units by only 2–3  fold, demonstrating the synergistic interaction between the bisbenzimidizole and A22.

**Figure 14:**
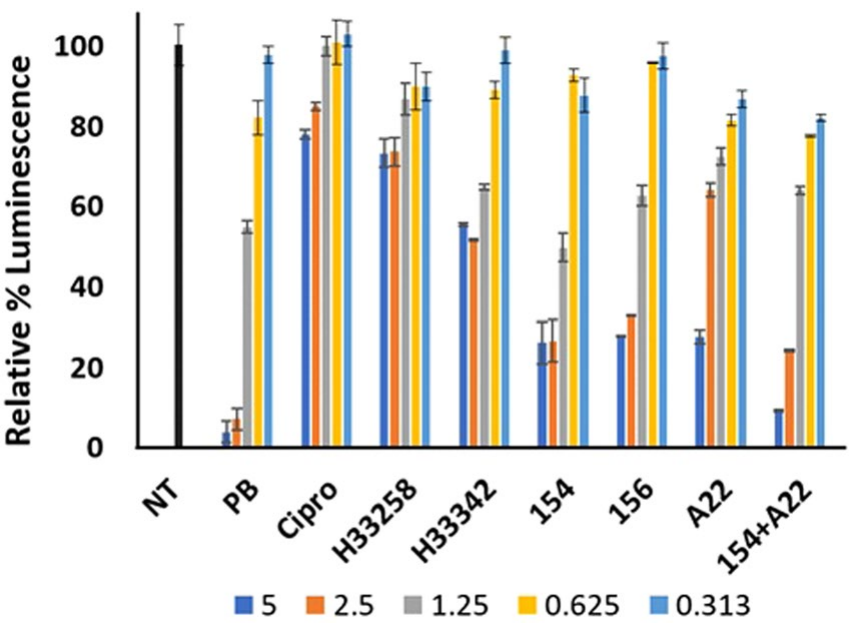
BacTitre Glo assay to ascertain cell viability in *E. coli* by inhibition of ATP synthesis by test compounds at a concentration range of 0.313–5 times the MIC for each compound (legend). Relative percent luminescence is proportional to cell viability. DPA 154 and 156 reduce ATP production and cell viability proportional to increasing concentration. DNA synthesis inhibitors and genotoxic agents (ciprofloxacin, Hoechst dyes) do not reduce cell viability to the same extent as compounds with postulated membrane active mechanisms (polymyxin-B, DPA 154/156, DPA 154/A22 combination). A22 reduced ATP synthesis to an extent comparable to DPA 154/156. DPA 154/A22 combination was superior in reducing cell viability compared to either DPA 154 or A22 alone and was similar to polymyxin-B. Exponential phase *E. coli* cultures were prepared with a 2-hour incubation with test compounds at 37 °C in microtiter plates. NT (No treatment), Cipro (Ciprofloxacin), H (Hoechst dye control), PB (Polymyxin-B), A22 (S-(3,4-dichlorobenzyl)isothiourea. Error bars represent two individual repeats with two duplicates.

ATP quantification supported culturable viability observations. When bacteria were challenged with a concentration range of test compounds there was a direct relationship between ATP production and test compound concentration (Fig. 14). ATP synthesis is directly related to cell viability and when the membrane is altered, the proton gradient is rapidly degraded with corresponding reduction in ATP synthesis. Agents that alter membrane permeability such as polymyxin-B exhibit a rapid collapse of the proton pump gradient we observe as the reduction of the percent luminescence relative to the untreated control. A similar reduction was observed with DPA 154. This contrasts with topoisomerase II inhibitor ciprofloxacin which does not induce membrane permeation, instead creating double-stranded DNA breakage which eventually leads to cell death^4,31^. While DPA 154 and A22 independently cause a reduction in ATP generation in *E. coli* and together induce a relative percent luminescence change similar to polymyxin-B, the same effect was not observed with Hoechst controls or ciprofloxacin within the two hour challenge period indicating a potent action of the bisbenzimidizole derivative alone in reducing bacterial cell viability.

### Membrane permeation studies

**Figure 15:**
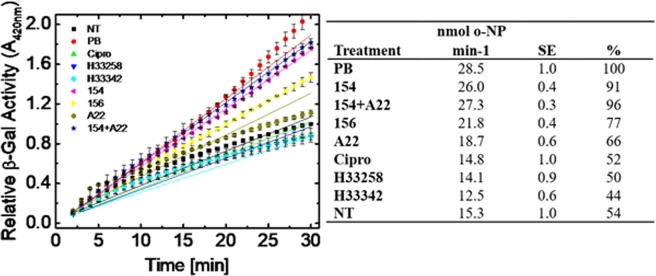
*E. coli* inner membrane permeabilization assay demonstrating linear membrane permeation capabilities of DPA 154/156 over time by release of cytoplasmic β-galactosidase and generation of *o-*nitrophenol at 420 nm. β-galactosidase activity was monitored over a period of 30 minutes exposure to each treatment of five times MIC of each compound. The amount of *o*-nitrophenol (*o*-NP) produced per minute was measured at 420 nm (ε = 4.5 mM^−1^ cm^−1^) over time at 37 °C with a saturating concentration of the substrate *o*-nitrophenyl galactoside (ONPG) at 3 mM (Table). Data are presented as nmols of *o*-nitrophenol (*o*-NP) produced per minute for bacteria incubated with and percent β-gal activity was determined relative to polymyxin B regarded as 100%. SE is the standard error of the of the mean of the slope of linear fit of data for three independent assays. DPA 154, 156, DPA 154/A22 combination, and polymyxin-B all induce noticeably greater release of cytoplasmic β-galactosidase indicating membrane permeabilization and membrane permeabilization increases as a function of time. DPA 154 is more effective at inducing membrane permeabilization than DPA 156. A22 alone has a moderate increase in membrane permeabilization and does not significantly increase membrane permeation in combination with DPA 154 as compared to DPA 154 alone, indicating there is no additive effect. Β-galactosidase (β-Gal) is a cytoplasmic enzyme that catalyzes the cleavage of lactose to glucose and galactose in lactose induced *E. coli*. When the inner membrane of *E. coli* is compromised β-Gal is released outside the cell and cleaves the substrate ONPG to the yellow product *o*-NP and galactose. Therefore, an increase in absorbance at A420 indicates membrane damage imparted by the test compound relative to untreated cells. NT (No treatment), Cipro (Ciprofloxacin), H (Hoechst dye control), PB (Polymyxin-B), A22 (S-(3,4-dichlorobenzyl)isothiourea. Error bars represent three individual repeats with two duplicates.

BCP concluded certain bisbenzimidizole derivitives exert effects of both membrane disrupting agents like polymyxin-B and DNA synthesis inhibitors such as ciprofloxacin. Previous studies have been performed that provide evidence for DNA synthesis by inhibition of bacterial topoisomerase I, but no such studies have validated the suggestions of a membrane active mechanism^8,9^. We observed that inner membrane disruption occurs when *E. coli* cells are exposed to DPA 154 or DPA 156 over time. Utilizing β-galactosidase leakage assay with cells exposed to DPA 154 or 156 we observe an immediate production of *o-*nitrophenol as comparable to polymyxin B (Fig. 15). Hoechst 33342, 33258 and ciprofloxacin do not result in an increase in β-galactose activity and are comparable to untreated cells, providing evidence that membrane disruption can be attributed to the addition of the terminal alkynyl linker to Hoechst 33258 derived compounds. Additionally, DPA 154 with the shorter terminal linker induces more β-galactosidase activity than DPA 156 at 30 minutes, corroborating with previous study and synergy assay results (Table S15) of DPA 154 being less effective than DPA 156^8^.

**Figure 16:**
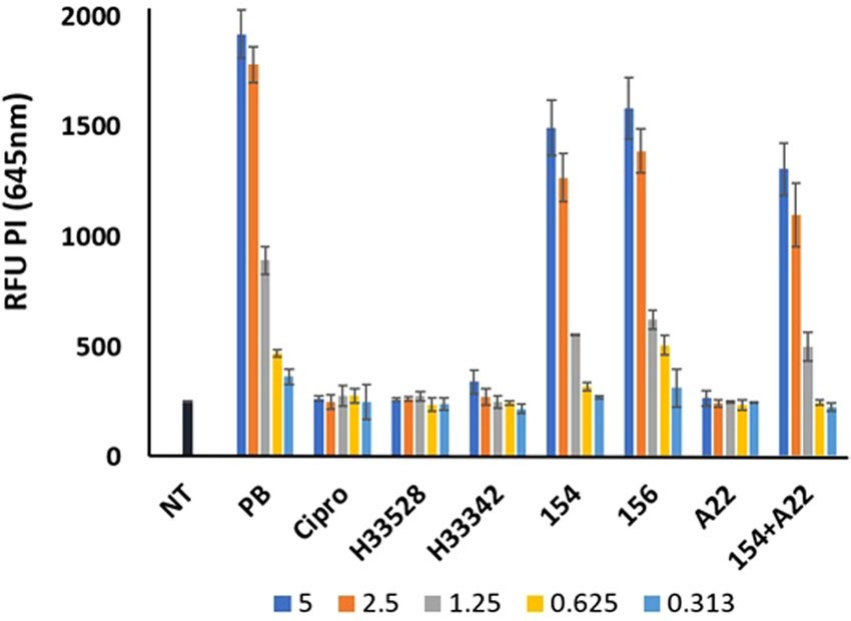
Propidium iodide (PI) staining assay of *E. coli* treated for two hours with varying concentrations of the MIC (5, 2.5, 1.25, 0.65, 0.313 times) demonstrating DPA 154 and DPA 156 increase membrane permeability as a function of concentration. Compounds with suspected membrane active mechanisms (DPA 154, 156, DPA 154/A22, polymyxin-B) induce a concentration dependent increase in fluorescence at 645 nm, indicating elevated intracellular PI. Non-membrane active compounds (Ciprofloxacin, Hoechst 33258/33342, A22) induce little significant PI uptake, suggesting DPA 154 and 156 are membrane active. mid-exponential phase culture of *E. coli* was used for antibiotic challenge. Relative fluorescence intensity units (RFU) at 645 nm is shown. PI fluorescence was measured (530ex, 645em) using a microplate reader after 15 minutes staining with PI. OD600 values of 0.25 for *E. coli* were used. Error bars represent 3 individual repeats with 2 replicates. The average values are given after subtracting the background of unbound PI. PI is a red fluorescent nucleic acid stain that intercalates with DNA and when bound, fluorescence is enhanced up to 30-fold upon penetration of cells with disrupted membranes and is excluded from viable cells. NT (No treatment), Cipro (Ciprofloxacin), H (Hoechst dye control), PB (Polymyxin-B), A22 (S-(3,4-dichlorobenzyl)isothiourea. Error bars represent three individual repeats with two duplicates.

Propidium iodide staining of *E. coli* at 2 hours and five times MIC also demonstrates the membrane disrupting effects of DPA 154 and DPA 156. Polmyxin-B, DPA 154, 156, and DPA 154/A22 combination iteratively increase the relative fluorescence units as a function of concentration (Fig. 16). Ciprofloxacin, Hoechst 33258, 33342, and A22 did not result in an increase in the permeability of *E. coli* cells as a function of concentration as Hoechst dyes freely diffuse through the membrane and recognize DNA and fluoroquinolones induce cell death via chromosome breakage and oxidative stress. Therefore, they do not introduce any membrane permeable phenotypes^5,16,20^.

Combined with the results of the inner membrane permeability assays we assert that DPA 154/156 compounds are membrane permeabilizing agents with activity dependent on both time and concentration of compound delivered, corroborating with BCP assay results suggestive of a mechanism of action similar to polymyxin-B with the appearance of lysed cells. Both alkynyl chain length and oxygen composition may be involved and further study is needed to model the critical interactions between linker lengths, heteroatoms, and the bacterial membrane to explain differences in membrane permeabilization. Lastly, A22 does not appear to directly affect membrane integrity where the DPA 154/A22 combination was not significantly more effective in membrane disruption than DPA 154 alone, suggesting that the mechanism of this particular synergistic interaction needs further study.

### The DPA 154 and A22 combination demonstrates synergistic against multi-drug resistant *Acinetobacter baumanii*

*A. baumanii* is a common cause of nosocomial infections and is frequently multi-drug resistant, necessitating development of novel antimicrobials^32,33^. We observe the DPA 154 / A22 combination was synergistic in the initial single point synergy assay. Therefore, checkerboard synergy assays were performed that compare DPA 154, DPA 156, Hoechst dyes, and the model DNA synthesis inhibitor ciprofloxacin in combination with A22 to validate the initial observation (Tables 3 and 4). Only DPA 154 demonstrated evidence of synergy in combination with A22 with an FICI < 0.5 in Gram-negative bacteria. In three independent assays we observed that the ciprofloxacin-resistant *A. baumanii* strain BC5 (MIC > 100 µM), a < 3 CFUs/mL reduction in cell culturability was observed with the DPA 154/A22 combination. Similar results were observed with antibiotic sensitive *E. coli* 25922 and the *E. coli* multidrug-resistant strain H4H (Table 2) a model pathogen that poses the challenge in antibiotic resistant urinary tract infections^34^.

**Table 3:** Synergy assay of critical combinations against *E. coli* and *A. baumanii*.

Bacterial Strain	MIC 154	MIC A22	FIC 154	FIC A22	FICI
*E. coli* 25922	32	64	4	8	**0.250**
*A. baumannii* BC5^a^	16	>64	4	4	**0.312**
*E. coli* H4H^a^	16	8	4	2	**0.500**
	**MIC H33258**	**MIC A22**	**FIC H33258**	**FIC A22**	**FICI**
*E. coli* 25922	>32	64	16	32	1.00
*E. coli* H4H	>32	8	4	4	0.625
*A. baumannii* BC5	>32	>64	8	32	0.75
	**MIC Cipro**	**MIC A22**	**FIC Cipro**	**FIC A22**	**FICI**
*A. baumannii* BC5	>32	>64	2	16	**0.312**
	**MIC 156**	**MIC A22**	**FIC 156**	**FIC A22**	**FICI**
*E. coli* 25922	>32	64	16	32	1
*E. coli* H4H	>32	8	4	4	0.625
*A. baumannii* BC5	32	>64	16	16	0.75

Synergy was evaluated between A22 and DPA156, H33258, ciprofloxacin or DPA156 by the checkerboard method following the broth microdilution as outlined by the Clinical Institute for Laboratory Standards. Bacteria were incubated overnight at 37 °C for 16 h for each assay in duplicate. Growth inhibition was determined by optical density at A595 nm. Cipro: ciprofloxacin, MIC: minimal inhibitory concentration, FIC: fractional inhibitory concentration and FICI: fractional inhibitory concentration index as calculated accordingly: FICI = FIC_1_/MBC_1_ + FIC_2_/MBC_2_. ^a^Mulitdrug-resistant strains. *A. baumannii* BC5 is a ciprofloxacin-resistant strain (MIC > 100 µM) while both *E. coli* strains are sensitive to ciprofloxacin (MIC 0.19–39 µM).

**Table 4:** Synergy assays and culturable enumeration against multidrug resistant *E. coli* and *A. baumanii*.

Bacterial Strain	MBC 154	MBC A22	FIC 154	FIC A22	FICI	Log CFU mL^−1^	SD CFU mL^−1^
*E. coli* 25922	>32	>64	4	8	**0.25**	3.2	0.2
*A. baumannii* BC5^a^	16	>64	4	2	**0.281**	BLD	—
*A. baumannii* BC5	16	>64	4	4	**0.312**	BLD	—
*A. baumannii* BC5	16	>64	2	8	**0.25**	2.8	0.2
	**MBC H33258**	**MBC A22**	**FIC H33258**	**FIC A22**	**FICI**	**LOG CFU ml** ^**−1**^	**SD CFU ml** ^**−1**^
*E. coli* H4H^a^	>32	>64	8	32	0.75	4.2	0.2
*E. coli* 25922	>32	>64	32	8	1.125	4.5	0.1
*A. baumannii* BC5	>32	>64	8	32	0.75	3.9	0.1
	**MBC Cipro**	**MBC A22**	**FIC Cipro**	**FIC A22**	**FICI**	**LOG CFU ml** ^**−1**^	**SD CFU ml** ^**−1**^
*A. baumannii* BC5	>32	64	8	32	0.75	3.6	0.1
	**MBC 156**	**MBC A22**	**FIC 156**	**FIC A22**	**FICI**	**LOG CFU ml** ^**−1**^	**SD CFU ml** ^**−1**^
*A. baumannii* BC5	>32	>64	16	32	1	4.7	0.1
*E. coli* 25922	>32	>64	32	64	2	9.3	0.3
*E. coli* H4H	>32	>64	32	64	2	9.6	0.1

Synergy was evaluated between A22 and DPA154, H33258, ciprofloxacin or DPA156 by the checkerboard method following the broth microdilution as outlined by the Clinical Institute for Laboratory Standards. DPA 154, but not 156 demonstrates synergy against MDR *A*. *baumanii*. Hoechst 33258, ciprofloxacin, and DPA 156 demonstrate no synergy. Bacteria were incubated overnight at 37 °C for 16 h for each assay in duplicate. Growth inhibition was determined by optical density at A595 nm and enumeration of colony-forming units (CFU) per mL was determined by spot plating with 10 µL from each well of serial 1:5 dilutions performed in triplicate. BLD: below the limit of detection (<2 LOG CFU/mL) where no colonies were observed; SD (standard deviation). Cipro: ciprofloxacin, MBC: minimal bactericidal concentration, FIC: fractional inhibitory concentration and FICI: fractional inhibitory concentration index as calculated accordingly: FICI = FIC_1_/MBC_1_ + FIC_2_/MBC_2_. ^a^Mulitdrug-resistant strains. *A. baumannii* BC5 is a ciprofloxacin-resistant strain (MIC > 100 µM) while both *E. coli* strains are sensitive to ciprofloxacin (MIC 0.19–39 µM). Spot plate follow-up for validation and checkerboards and additional replicate synergy data are available in Supplemental Material (Figs S21–S23).

### Possible mechanisms for synergistic combination of 154 and A22

While A22 alone was not inhibitory against the *E. coli* strain used here the combination with DPA 154 was potent. A22 (S-3,4 dichlorobenzyl isothiourea) is a cytoskeletal inhibitor that exerts its antibiotic effect by inhibiting the polymerization of actin-like fibers built by *E. coli* MreB through disruption of phosphate release from the enzyme^35,36^. A22 binds competitively to the nucleotide-binding site of MreB, causing cells to collapse into ovoid cells through a mechanism not fully explained^16^. One potential explanation posits MreB homopolymers deliver peptidoglycan building complexes such as penicillin-binding proteins (PBPs) to the cell wall, and dysregulation of the actin-like cytoskeleton MreB causes the bacterial cell to be unable to organize longitudinal peptidoglycan synthesis. This contributes to a spheroplastic phenotype seen in previous studies^37^.

The macroscale interaction between DPA 154 and A22 is also not clear, but one potential mechanism is the reduced ability for biofilm formation of A22-induced ovoid cells. The morphology shift from rod to coccus has been shown to be detrimental for biofilm, surface, and epithelial binding, so reduction in the efficacy of biofilm adherence may allow for increased compound activity^38,39^. This idea is further supported by the evidence that MreB is only required for cell shape and not chromosome partitioning, indicating the induced shape is a critical aspect of the antimicrobial effect of A22^40^. Furthermore, inhibition of MreB may disrupt the mediation of peptidoglycan synthesis through the interaction of MreBCD and penicillin-binding proteins. If this is the case, part of the synergistic effect may come from dual inhibition of the ultrastructure in the periplasmic space and the outer membrane-active effects of the DPA 154 hydrophobic linker^41^. Alternatively, formation of a topoisomerase 1A ternary complex may compromise cell membranes by inhibition of DNA synthesis^1^. More studies are needed to study the interplay of both of these mechanisms of action.

## Conclusion

In summary, we show here much improved Gram-negative coverage of alkynyl bisbenzimidazoles by combination treatment with A22 and determined a dual mechanism of action by inhibition of DNA synthesis and membrane integrity. Using BCP we showed alkynyl bisbenzimidazoles induce spheroplastic phenotypes and membrane lysis consistent with membrane active compounds like nisin and polymyxin-B and alter DNA morphology similar to the fluoroquinolones. Synergy screens and time-kill assays revealed the MreB inhibitor A22 to be highly synergistic with DPA 154, the compound which previously had the best Gram-positive activity, against many WHO high priority pathogens including multidrug-resistant *A. baumanii* and *E. coli*. Synergy studies also revealed the Gram-negative outer membrane to be a major obstacle for efficacy of alkynyl bisbenzimidazoles alone. Alkynyl bisbenzimidazoles are attractive candidates for development as versatile antibiotics to help overcome rampant antibiotic resistance. Further studies are ongoing to fully understand the synergistic interactions between A22 and DPA 154, and how this improves on greater Gram negative coverage.

## Methodology

### Synthesis of DPA compounds

Synthesis, spectral data, and purity of DPA compounds have been reported elsewhere^8,9^.

### Strains

Fluorescence imaging was performed using *E. coli* 25922 (ATCC) and *B. subtilis* 6051 (BEI Resources) as model organisms. The following strains used for evaluation of synergistic combinations were *E. faecium* BM4105-RF (BEI), *E. faecalis* 29121 (ATCC), *S. aureus* 25923 (ATCC), MRSA 33591 (ATCC), *K. pneumoniae* NR15410 (BEI), *K. pneumoniae* 1332 ARLG (Antibiotic Leadership Group), *A. baumanii* BC5 (BEI), *A. baumanii* 19606 (ATCC), *P. aeruginosa* 27853 (ATCC), *E. cloacae* 13047 (ATCC), *E. coli* 25922 (ATCC), *E. coli* H4H and (BEI). Bacteria were routinely maintained on tryptic soy agar or broth. Minimal inhibitory concentration of antibiotics and test compounds were determined by the broth microdilution assay using the guidelines of the Clinical Laboratory Standards Institute.

### Chemicals

Twelve antibiotics affecting the five major biosynthetic pathways classes (DNA synthesis, RNA synthesis, protein synthesis, cell wall synthesis, and fatty acid synthesis) were included in the cytological profiling analysis. A list of antibiotics used in this study along with mechanism of action are available in Supplemental Material Table S6 Antibiotics stocks solutions were prepared at 50X in DMSO or water and diluted 10-fold for a final concentration of five times the MIC in flat bottom 96 well plates. Ten compounds were selected for synergy assays (Table S16). Chemicals were purchased from Sigma-Aldrich Inc. or Fisher-Scientific and prepared according to the manufacturers indications. Dyes used were SYTOX Green (ThermoFisher), FM4-64 (ThermoFisher), and DAPI (Sigma-Aldrich Inc.).

### Fluorescence microscopy

Methods for cytological profiling follow the procedure established by *Nonejuie et al*.^11^. Preparation of bacteria for fluorescence microscopy included growth of bacteria to mid-log phase in Luria-Bertani (LB) broth and diluted in LB followed by treatment with five times the MIC of each drug with 90 µL log-phase cells (~10^6^ cells/ well) aliquoted to microtiter plates at 37 °C for 3 hours. Challenged bacteria were retrieved after 3 hours and stained with 10 µL of a 10x solution of dye mix dissolved in 100 mM Tris base for a final concentration of each dye at 1 µg/mL FM4-64 membrane-staining dye, 2 µg/mL DAPI nuclear material-staining dye, and 0.5 µM SYTOX GREEN cell-permeation assay agent. Cells were stained for 15 min at room temperature in the dark prior to microscopy. Dilutions of stained cell suspension were performed as needed into 96 well black walled clear flat-bottomed fluorescence microscopy plates prior to microscopy to achieve one cell layer for optimal imaging and analysis. Each treatment was analyzed with triplicate wells and 4 fields per wavelength per well. Exposure time for each wavelength was held consistent across all wells. Imaging was performed using the GE IN Cell Analyzer 2500HS system. All fluorescent stains and media were obtained from Fisher Scientific. 96 well microplates for imaging were purchased from Greiner Bio One.

### Quantitative analysis

Cell/DNA segmentation was analyzed by the GE IN Cell 2500HS developer packet and ImageJ v150i using drawn polygons to determine the area (µm^2^), length (µm) diameter (µm), form factor, and perimeter (µm) of both the cell membrane and DNA. Cellular form factor was used as an approximation of the circularity of each cell. Additionally, weighted moment of inertia was used as a measurement of cell movement. Additional counts of SYTOX GREEN-permeable cells, nucleoid count, and Measures of SYTOX GREEN and DAPI levels were defined as the fluorescence density x area. Complete definition of each variable may be found in Supplemental Material (Table S9). Graphical representations of this data were generated in Minitab 18.

### Principle component analysis

Data was retrieved from ImageJ output in.xlsx format. Cytological profiling data was taken from every bacterium if possible (*B. subtilis* 1018 < n < 5157; *E. coli* 106 < n < 1221); For a full list of bacterial count by treatment see Table S14. Quantitative data was averaged to produce a mean for each antibiotic. PCA utilizing Spearman’s rank correlation for evaluation of monotonic variables was performed from this averaged data. Visualization of principal components was built using XL-STAT 2018.1. The biplots used for visualizing principal component data was a n/p correlation biplot with vectors and labels. Scree plots describing contribution to variability were generated and may be found in Supplemental Material (Figs S1–S4). BCP assay plate also included unrelated compounds used for a different study analyzed in parallel and were therefore removed from this analysis. No compounds in this study were removed from analysis.

### Clustering

Agglomerative hierarchical clustering with associated heat-map was generated using the nominal variables PC1, PC2, and PC3 (principal components from XL-STAT output) in the ClustVis web application https://biit.cs.ut.ee/clustvis/.42 Clustering used Euclidean distance and the agglomeration method of choice was Ward’s Method. Data was reduced, centered, and assigned by columns. Missing data was removed. BCP assay plate also included unrelated compounds used for a different study analyzed in parallel. Some compounds not studied in this paper were fluorescent and interfered with absorption data and thus had incomplete data and were removed from analysis. No compounds in this study were removed from analysis.

### Minimal inhibitory concentration determination

Minimum inhibitory concentrations (MICs) were determined by microbroth dilution method via the guidelines of the Clinical Laboratory Standard Institute^42^. Bacterial cultures were inoculated in tryptic soy broth and grown to exponential phase on a shaker at 37 °C for 2–3 hours. Exponential-phase cultures were then diluted 1:10000x in MHII medium and 90 µL cell suspension was added to 96 well plates containing 10 µL of a 10x solution each antibiotic at each serial 1:2 dilution. Plates were incubated overnight at 37 °C and analyzed via optical density (OD 595 nm) for determination of MIC. The percent growth inhibition was determined for replicate assays as follows:

$$ \% \,Growth\,Inhibition=100-100x\frac{{A}_{compound}-{A}_{background}}{{A}_{control}-{A}_{background}}$$

### Synergy assays

Synergy assays were initially performed at a single point concentration of test compound (dependent on the MIC value for each compound) and a 1:2 serial dilution of DPA compound using the microbroth dilution method. Hoechst acids were diluted from 64–0.5 µM and combined with equal volume of a static concentration of synergy compound. Percent inhibition values were calculated using the same method as MIC determination.

Checkerboard dilutions were performed to verify the results from the single point synergy assays with significant results. Perpendicular dilutions of equal volume were added to a 96 well plate and incubated with MHII agar inoculated with strains of choice. Fractional inhibitory concentrations were calculated using the equation $(\frac{FIC\,Hoechst\,acid\,in\,combination}{MIC\,Hoechst\,acid\,alone})+(\frac{FIC\,synergy\,compound\,in\,combination}{MIC\,synergy\,compound\,alone})$ = Fractional Inhibitory Concentration Index (FICI). FICI defines Synergy ≤0.5, Indifference 0.5 < x < 4, and antagonism ≥4.

### Time-Kill assay

*E. coli* ATCC 25922 was grown to logarithmic phase and diluted to ~5 × 10^6^ colony-forming units and exposed to each of the following in MHII broth: two times MIC norfloxacin, 2 µM Hoechst 33342 + 4 µM A22, 4 µM Hoechst 33342 + 8 µM A22, 8 µM Hoechst 33342 + 16 µM A22, and 8 µM DPA 154 + 16 µM A22. 100 µL aliquots from each treatment were collected after 0, 1, 2, 4, 6, 8, and 24 hours incubation on a shaker at 37 °C and serially diluted before plating on TSA plates to determine colony forming units. Synergy was defined as ≥2-fold reduction in CFU/mL relative to the untreated control.

### Determination of bacterial cell viability by CFUs

The single plate serial dilution spotting technique was used to enumerate colony-forming units^43^. Briefly, 10 µL of serial 1:10 dilutions of each treatment in duplicate was spot plated to tryptic soy agar plates. After absorption of the bacterial suspension plates were inverted and incubated at 37 °C for no more than 16 h before determining CFU/mL.

### Determination of bacterial cell viability by BacTitre Glo assay

Determination of the viability of bacteria by ATP measurement^44^. Bacteria were challenged with test compound in a black-walled 96 welled microtiter plate for 16 h at 37 °C. After incubation the BacTiter-Glo assay (Promega) was used according to the manufacturer’s guidelines. Briefly, an equal volume of BacTiter-Glo was added to the wells of the antibiotic challenge microtiter plate. Plates were incubated for 5 min at room temperature on a shaker. Luminescence was measured using a TECAN-PRO plate reader. The average percent luminescence was calculated after background fluorescence subtraction. Assays were performed in duplicate on 2 separate occasions. Data are presented as the relative percent luminescence relative to the untreated control.

### SYTOX Green fluorescence assay

*E. coli* ATCC 25922 cultures were initiated by inoculation of 5 colonies to 25 mL MH II broth incubated at 37 °C with shaking for 2 h to achieve log-phase cells. After incubation, cultures were diluted in MH II to an OD600 of 0.1 for a cell density of 10^9^ cells/mL. For antibiotic challenge, 90 μL of log-phase culture was added to each well of a 96 well microtiter plate containing 10 μL of 50–0.1x the MIC of each compound for a final cell number of 10^8^ and relative MIC range of 5–0.01 times. Culture plates then were incubated for 3 h at 37 °C. After antibiotic challenge, 40 μL from each well was transferred to a clear-bottom black-walled 96 well plate and 10 uL of a 10 μM solution of SYTOX Green (in 100 mM TRIS base) was added to each well for a final of 2 μM dye. Plates were incubated at 37 °C in the dark for 30 min. Fluorescence intensity was measured using a TECAN infinite M1000 PRO plate reader using a bottom reading with 485 nm excitation and 535 nm emission wavelengths. Culturable enumeration was determined after dilution (10^−1^–10^−8^) using the spot plate method by plating 10 μL from each well using a multichannel pipettor onto tryptic soy agar square plates. Plates were incubated 24 h at 37 °C before counting colonies.

### β-galactosidase assay for inner membrane permeabilization

Membrane permeabilization was determined by measuring the release of cytoplasmic β-galactosidase activity from *E. coli* into the culture medium using the substrate ONPG as measured over the time of exposure to test compounds (2 h)^45^. Exponential-phase bacteria grown in nutrient broth containing 2% lactose were harvested by centrifugation and washed two times in 0.85% saline solution. The final cell suspension in saline solution was adjusted to obtain an OD_600_ of 0.8. One hundred microliters of bacterial suspension were added to the wells of a 96 well plate containing test compound at five times the MIC (determined previously) and the substrate ONPG was added at a final concentration of 30 mM. The plates were immediately read to measure the increase in A_420_ (*o*-NP production) over a period of 2 h using the plate reader on kinetic cycle. Assays were performed in duplicate on three separate occasions.

### Propidium iodide assay for membrane permeabilization

Propidium iodide (PI) is a membrane impermeable red fluorescent nucleic acid stain that intercalates with DNA and when bound fluorescence is enhanced up to 30-fold and penetrates cells with disrupted membranes and is excluded from viable cells^46^. Exponential-phase bacteria grown in nutrient broth were harvested by centrifugation and washed two times in 0.85% saline solution. The final cell suspension in saline solution was adjusted to obtain an OD_600_ of 0.1 (~10^9^ CFU/mL). Ninety microliters of bacterial suspension were added to each well of a black 96-well clear bottom plate the wells containing 10 μL test compound at 50 times the MIC after which 50 μL of 15 μM propidium iodide was added per well before incubating additional 15 minutes on an orbital shaker in the dark. After incubation with PI fluorescence intensity was measured using a TECAN-PRO plate reader with excitation at 530 nm and emission at 645 nm using a bottom reading. Assays were conducted in duplicate on three separate occasions. Data are presented as the average relative fluorescence units relative to the untreated control bacterial suspension.

## Supplementary information

Supporting Info
