# Supplementary material for: Gram-negative synergy and mechanism of action of alkynyl bisbenzimidazoles
Source: Sci Rep. 2019 Oct 2;9:14171. doi: 10.1038/s41598-019-48898-4 (PMC6775084; doi:10.1038/s41598-019-48898-4)
Supplement: Supplementary file 1 — Supporting Info [file 41598_2019_48898_MOESM1_ESM.pdf]

Gram-negative synergy and mechanism of action of alkynyl bisbenzimidazoles.

Jordan Chamberlin[1], Sandra Story[2], Nihar Ranjan[1#], Geoffrey Chesser[1], Dev P. Arya[1,2*]

[1] Department of Chemistry, Clemson University, Clemson, 29631, USA

[2] NUBAD LLC, Greenville, 29605, USA

[1#] National Institute of Pharmaceutical Education and Research (NIPER) Raebareli , Lucknow, Uttar Pradesh, India

*Address correspondence to Dev P. Arya, dparya@clemson.edu

Supplemental Data

Morphology	Description	Mechanism	Phenotype
Spheroplast [Not Pictured]	Large, spherical, osmotically sensitive cells	Inhibition of ribosomal synthesis of proteins related to peptidoglycan synthesis	
Filamentation (moderate) [A]	Moderate (1-1.6x) lengthening of bacterial cells	Unknown mechanism.	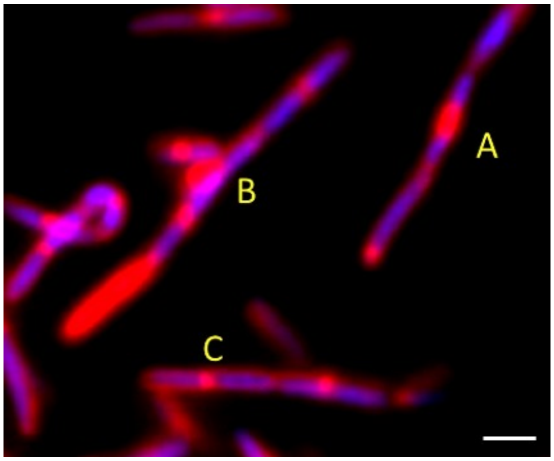 **Kanamycin**
Bulging [B]	Localized Protrusion composed of all envelope components	Accumulation of peptidoglycan cross-links defects	
Peptidoglycan thickening (Gram + only) [C]	Septal and peripheral peptidoglycan thickening	Inhibition of synthesis of peptidoglycan hydrolases	

Table S1. Expected morphologies for bacteria treated with protein synthesis inhibitors.[1-3] Scale bar 1 μM. Pictured is *B. subtilis*.

Morphology	Description	Mechanism	Phenotype
Spheroplast [A]	Large, spherical, osmotically active cells	loss of peptidoglycan layer by inhibition of PBP1a and 1b	
Filamentation (long) [B]	Extensive lengthening of rod-shaped cells (>2x)	Unequal lengthening of lateral cell wall due to inhibition of PBP3	
Mid-section Swelling [C]	Localized bilateral swelling with retainment of osmotic stability	Simultaneous inhibition of PBP2 and PBP3	
Bulging [Not Pictured]	Localized unilateral swelling and are osmotically sensitive	Accumulation of peptidoglycan cross-link defects leading to pore formation	
Peptidoglycan Thickening [D]	Septal and peripheral peptidoglycan thickening	Inhibition of synthesis of peptidoglycan hydrolases	
Leakage / lysate [E]	Leaked cellular contents (DNA)	Loss of cell integrity due to cell wall destruction	
Ghost cells [F]	Empty cell envelopes staining completely red with FM4-64	Various mechanisms including activation of lysis gene *E*	

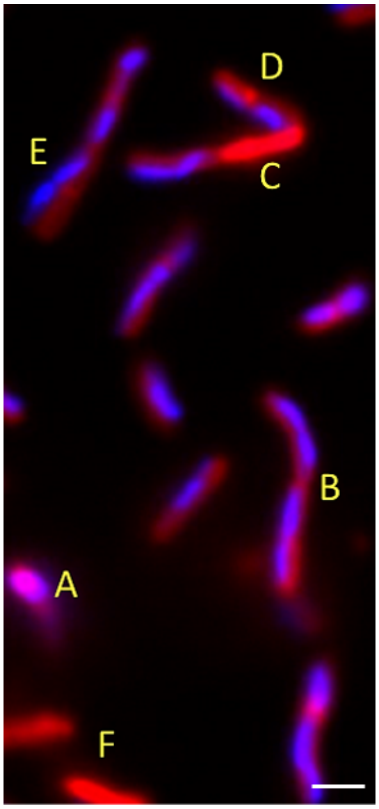

Ampicillin

Table S2. Expected morphologies for bacteria treated with cell wall synthesis inhibitors.[2-5] Scale bar 1 μM. Pictured is *B. subtilis*.

Morphology	Description	Mechanism	Phenotype
Spheroplast [A]	Large, spherical, osmotically sensitive cells	Supra inhibitory concentrations inhibit RNA and protein synthesis, loss of peptidoglycan layer	
Filamentation (long) [B]	Extensive lengthening of rod-shaped cells (>2x)	Induction of bacterial SOS response to DNA strand breakage	
Leakage / lysate [Not Pictured]	Leaked cellular contents (DNA)	Loss of cell integrity due to cell wall destruction	
Ghost cells [C]	Empty cell envelopes staining completely red with FM4-64	Various mechanisms including activation of lysis gene *E*	

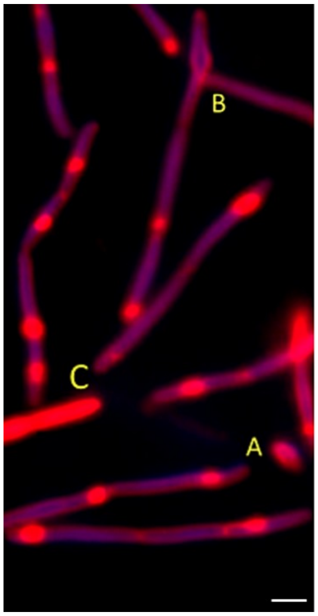

Ciprofloxacin
B. subtilis

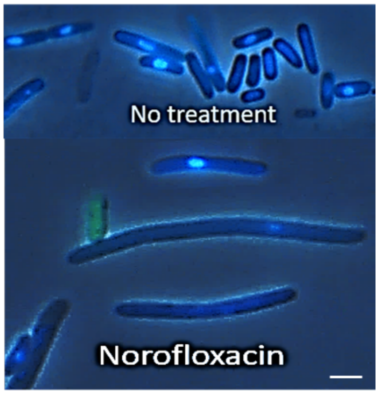

E. coli

Table S3. Expected morphologies for bacteria treated with DNA synthesis inhibitors.[2, 5-7] Pictured is *B. subtilis* (top) *and E. coli* (bottom). Scale bar 1 μM.

Morphology	Description	Mechanism	Phenotype
Filamentation (moderate) [A]	Moderate (1-1.6x) lengthening of bacterial cells	Unproposed mechanism	
Bulging [B]	Multiple localized unilateral protrusions	Nondescript disturbance of peptidoglycan – cell membrane biosynthetic relationship	
Leakage / lysate [Not Pictured]	Leaked cellular contents (DNA)	Loss of cell integrity due to cell wall destruction	
Ghost cells [C]	Empty cell envelopes staining completely red with FM4-64	Various mechanisms including activation of lysis gene *E*	

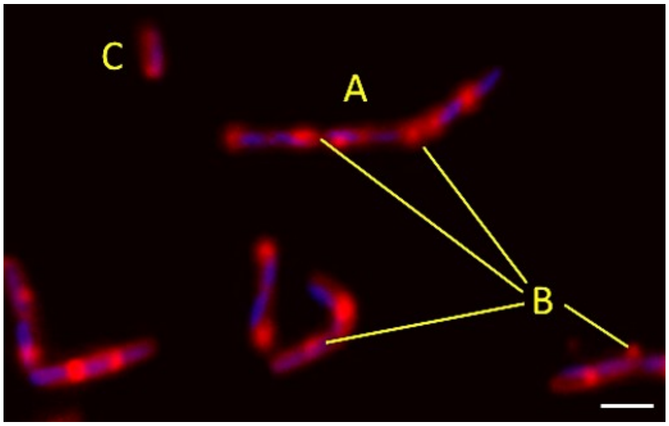

Rifampicin

Table S4. Expected morphologies for bacteria treated with RNA synthesis inhibitors.[2, 5, 8]
Scale bar 1 μM. Pictured is *B. subtilis*.

Morphology	Description	Mechanism	Phenotype
Filamentation (Moderate) [A]	Moderate (1-1.6x) lengthening of bacterial cells	Unproposed mechanism	
Bulging [Not Pictured]	Multiple localized unilateral protrusions	Chemical intercalation of membrane disruptor causes local mechanical increase in membrane surface area.	
Leakage/ lysate [B]	Leaked cellular contents, breakdown of membrane integrity	Destruction of outer membrane integrity by binding to lipids etc.	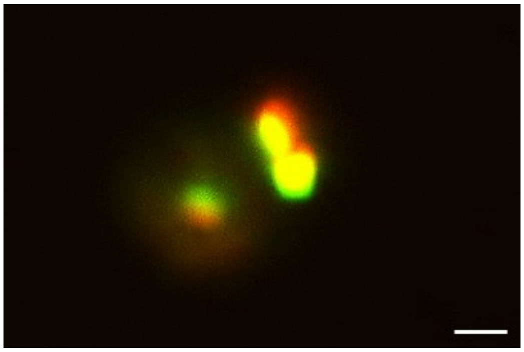 **Polymyxin B [E. coli]**

Table S5. Expected morphologies for bacteria treated with membrane-active agents.[2, 9] Scale bar 1 μM. Pictured is *B. subtilis*.

Antibiotics for use in Cytological profiling

Antibiotic	Mechanism of action[10-12]
Vancomycin	Cell wall synthesis; D-Ala-D-Ala binding
Ampicillin	Cell wall synthesis; PBPs
Ciprofloxacin	DNA Synthesis; DNA gyrase
Nalidixic acid	DNA Synthesis; DNA gyrase
Tobramycin	Protein synthesis; mistranslation
Neomycin	Protein synthesis; mistranslation
Kanamycin	Protein synthesis; mistranslation
Linezolid	Protein synthesis; Initiation complex formation inhibition
Tetracycline	Protein synthesis; tRNA binding
Rifampicin	RNA synthesis; DNA-dependent transcription
Nisin	Membrane-active; Lipid II binding and pore formation.
Polymyxin B	Membrane active; LPS binding and membrane integrity destruction.

Table S6. Antibiotics used in cytological profiling in this study. Compounds are representative of the five major inhibitory classes (DNA synthesis, RNA synthesis, protein synthesis, cell wall synthesis, membrane-active compounds). Alternative antibiotics have been described in *Nonejuie et al.*[13]

Escherichia coli

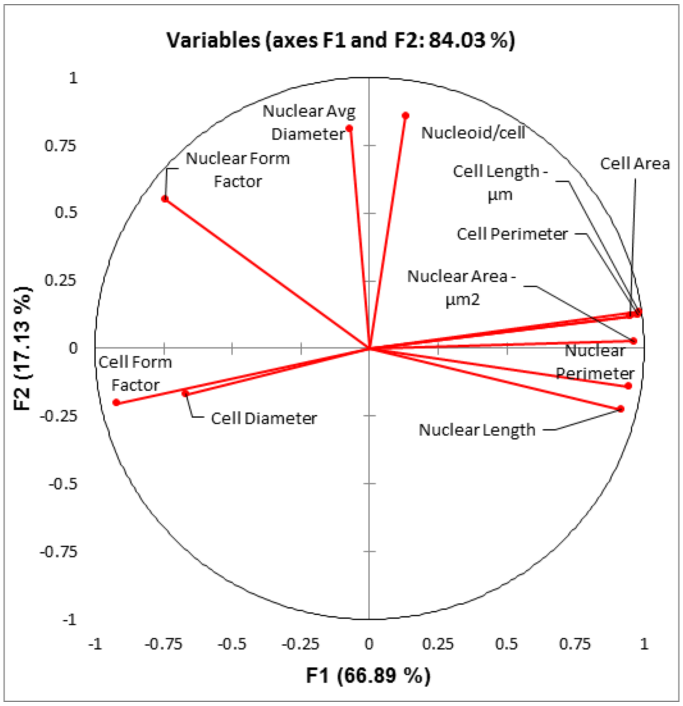

Figure S1. Variable Axes chart representing correlations between variables and principal components from this compound library treated *E. coli*.

	F1	F2	F3
Cell Area - µm2	12.264	0.754	1.675
Cell Diameter - µm	6.061	1.539	39.690
Cell Form Factor	11.506	2.163	4.880
Cell Length - µm	13.120	0.987	0.011
Cell Perimeter - µm	12.992	0.812	0.773
Nuclear Area - µm2	12.654	0.035	4.267
Nuclear Avg Diameter - µm	0.066	34.927	27.704
Nucleoid/cell	0.234	39.025	6.096
Nuclear Form Factor	7.478	15.995	0.255
Nuclear Length - µm	11.476	2.701	7.472
Nuclear Perimeter - µm	12.151	1.063	7.176

Table S7. Percent contributions of each variable from this compound library for *E. coli*.

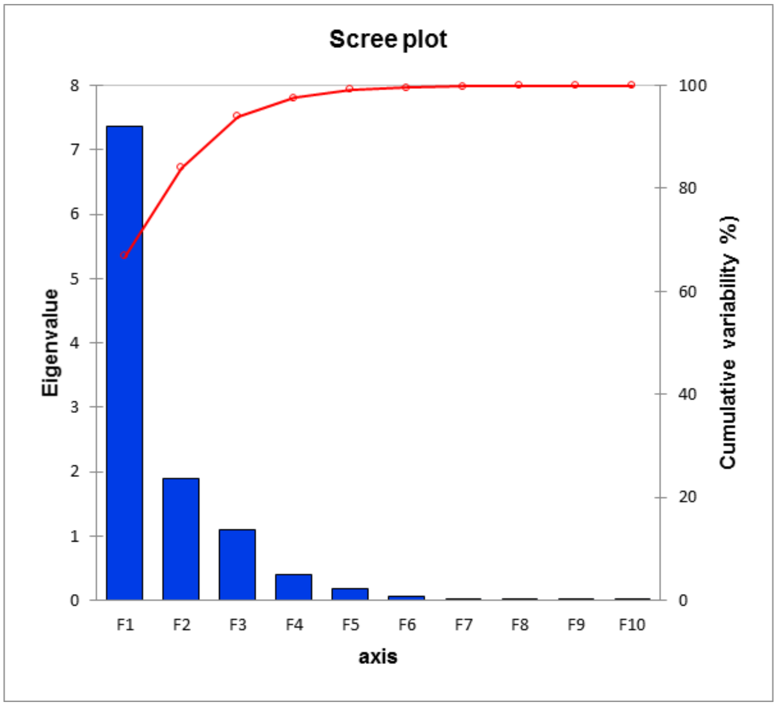

Figure S2. Scree plot of eigenvalue and cumulative variability of all principal components for this compound library for *E. coli*.

B. subtilis

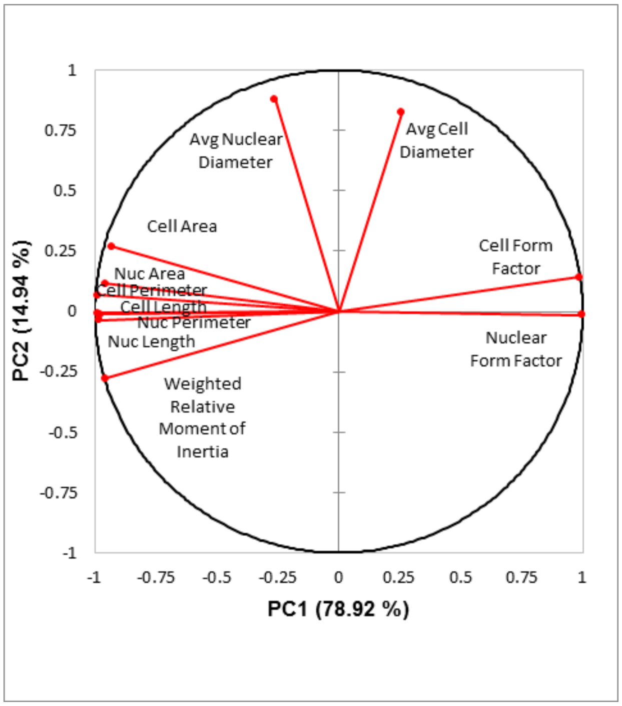

Figure S3. Variable Axes chart representing correlations between variables and principal components from this compound library treated *B. subtilis*.

	PC1	PC2	PC3
Cell Area - µm2	10.032	4.398	7.912
Cell Avg Diameter - µm	0.796	47.036	25.833
Cell Form Factor	11.171	1.229	0.352
Cell Length - µm	11.317	0.003	1.748
Cell Perimeter - µm	11.218	0.275	2.664
Weighted Relative Moment of Inertia	10.527	4.715	0.811
Nuclear Area - µm2	10.593	0.824	9.199
Nuclear Avg Diameter - µm	0.770	41.419	42.870
Nuclear Form Factor	11.407	0.013	0.030
Nuclear Length - µm	11.130	0.079	3.628
Nuclear Perimeter - µm	11.038	0.010	4.954

Table S8. Percent contributions of each variable from this compound library for *B. subtilis*.

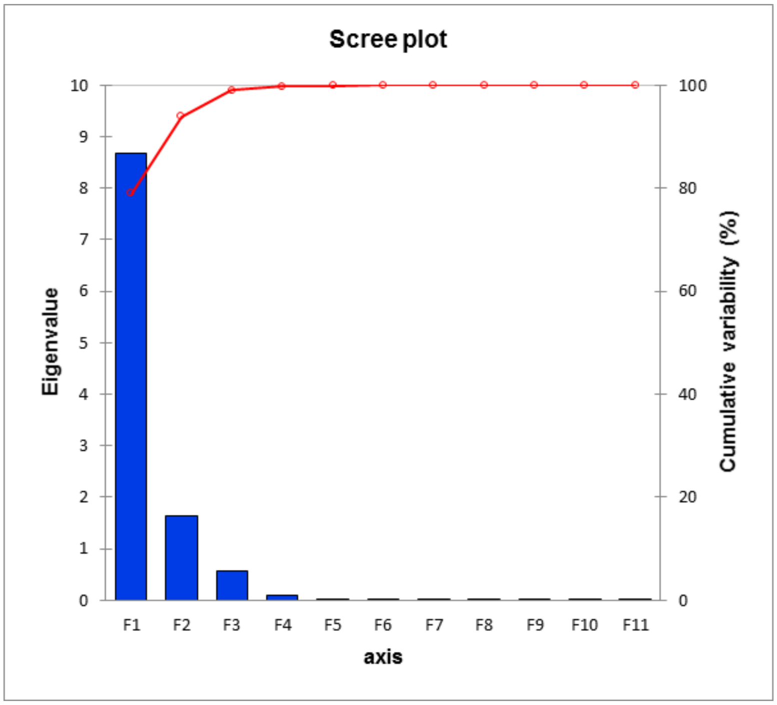

Figure S4. Scree plot of eigenvalue and cumulative variability of all principal components for this compound library *B. subtilis*.

Measurement	Parameters
**Area**	Calculated area by counting pixels inside target outline borders. Pixels belonging to the border itself are not included.
**Average Diameter**	Mean internal distance perpendicular to the curved chord.
**Count**	The number of targets contained within the region of interest.
**Form Factor**	Standard estimate of circularity that relates perimeter length to area. The more convoluted (and longer) the perimeter, the less circular the target. Varies from 0 to 1, with 1 being a perfect circle.
**Length**	Length is the maximum distance across a target and it is a measurement that is allowed to cross target boundaries. In an S-shaped target, length is the straight line distance from tip to tip of the S.
**Perimeter**	Length of target's outer boundary. Local, curvature-sensitive smoothing used during perimeter calculation with IN Cell developer Toolbox reduces excess smoothing due to artifacts.
**Weighted Relative Moment Of inertia**	Index of the homogeneity of gray levels within a circular target. A value of 1 indicates the target is relatively homogeneous. If >1, the target has a higher proportion of bright pixels in its center. If <1, the target has a higher proportion of bright pixels around its perimeter.
**Density**	Mean density value of the pixels contained within the target outline. **Levels** – Gray Levels. Uncalibrated intensity unit for IN Cell images, the higher the value, the brighter the pixel.
**DxA**	Density x Area. Mean density (in current density unit) within the target outline multiplied by its area. Estimates total signal intensity associated with a given target.
**Permeable Cells**	Defined as sum count of green cells divided by sum count of all cells

Table S9. Parameters for measurements recorded in cytological profiling and methods for replication.

B. subtilis

	**Area - μm^2**	**Diameter - µm**	**Form Factor**	**Length - µm**	**Perimeter - µm**
DPA154	5.091	1.57	0.861	3.467	8.445
DPA156	5.417	1.624	0.869	3.556	8.73
Ampicillin	6.927	1.538	0.72	5.074	11.903
Ciprofloxacin	7.973	1.361	0.694	6.098	14.007
Kanamycin	9.253	1.505	0.661	6.553	15.284
Linezolid	7.533	1.568	0.707	5.278	12.329
Nalidixic Acid	8.829	1.402	0.638	6.83	15.626
Neomycin	8.621	1.504	0.651	6.289	14.58
Nisin	4.076	1.589	0.924	2.88	7.256
Polymyxin B	5.763	1.677	0.843	3.825	9.345
Rifampicin	7.281	1.589	0.709	5.167	12.104
Tetracycline	7.299	1.501	0.713	5.213	12.203
Tobramycin	7.211	1.514	0.704	5.2	12.187
Vancomycin	7.047	1.577	0.719	5.066	11.817
Untreated	1.076	1.372	0.963	1.447	3.681

Table S10. Cell morphology parameters used for PCA and AHC

	**Area - μm^2**	**Diameter - µm**	**Form Factor**	**Length - µm**	**Perimeter - µm**
DPA154	1.139	0.695	0.911	1.592	3.868
DPA156	1.15	0.708	0.949	1.436	3.568
Ampicillin	1.864	0.785	0.761	2.571	5.729
Ciprofloxacin	1.515	0.415	0.626	2.871	5.974
Kanamycin	2.015	0.548	0.609	3.226	6.835
Linezolid	1.795	0.637	0.721	2.649	5.785
Nalidixic Acid	1.746	0.442	0.582	3.146	6.557
Neomycin	2.104	0.593	0.623	3.231	6.861
Nisin	0.47	0.416	0.971	0.991	2.394
Polymyxin B	0.639	0.431	0.873	1.307	3.007
Rifampicin	1.925	0.64	0.694	2.84	6.214
Tetracycline	1.873	0.61	0.681	2.823	6.114
Tobramycin	1.872	0.614	0.689	2.805	6.086
Vancomycin	1.85	0.68	0.72	2.676	5.888
Untreated	0.384	0.365	1.012	0.896	2.127

Table S11. DNA Morphology parameters used for PCA and AHC.

E. coli

	Area - μm^2	Diameter - µm	Form Factor	Length - µm	Perimeter - µm
DPA 154	4.305	1.262	0.683	3.989	9.112
DPA 156	3.806	1.373	0.804	3.306	7.775
Ampicillin	7.892	1.164	0.492	7.911	17.046
Ciprofloxacin	3.737	1.322	0.770	3.391	8.066
Kanamycin	4.160	1.170	0.669	4.030	9.231
Linezolid	3.879	1.242	0.725	3.658	8.411
Nalidixic Acid	6.742	1.179	0.478	6.590	14.323
Neomycin	3.902	1.166	0.694	3.789	8.686
Nisin	3.937	1.258	0.728	3.674	8.459
Polymyxin-B	2.548	1.296	0.897	2.402	5.944
Rifampicin	4.226	1.245	0.678	4.039	9.142
Tetracycline	3.822	1.254	0.745	3.542	8.255
Tobramycin	3.735	1.247	0.718	3.578	8.308
Vancomycin	4.291	1.266	0.690	4.018	9.114
Untreated	4.120	1.226	0.678	4.000	9.129

Table S12. Cell morphology parameters used for PCA and AHC.

	Area - μm^2	Diameter - µm	Form Factor	Length - µm	Perimeter - µm
DPA 154	3.163	1.187	0.745	3.215	7.526
DPA 156	3.302	1.275	0.788	3.060	7.431
Ampicillin	9.336	1.171	0.407	9.020	19.083
Ciprofloxacin	3.200	1.223	0.781	3.113	7.320
Kanamycin	3.739	1.210	0.718	3.605	8.267
Linezolid	3.496	1.254	0.769	3.344	7.675
Nalidixic Acid	6.265	1.209	0.518	6.107	13.168
Neomycin	3.650	1.234	0.741	3.514	8.039
Nisin	3.889	1.254	0.722	3.678	8.409
Polymyxin-B	2.374	0.948	0.704	2.913	6.569
Rifampicin	4.066	1.261	0.721	3.777	8.598
Tetracycline	3.516	1.256	0.771	3.297	7.691
Tobramycin	3.579	1.239	0.754	3.375	7.998
Vancomycin	4.020	1.289	0.736	3.691	8.439
Untreated	3.757	1.190	0.713	3.696	8.365

Table S13. Cell morphology parameters used for PCA and AHC.

	B. subtilis	E. coli
**TET**	4261	550
**CIP**	5008	214
**KAN**	4585	518
**NAL**	5157	302
**AMK**	4260	501
**RIF**	2874	1221
**NEO**	3644	507
**AMP**	1414	196
**TOB**	3200	239
**VAN**	1699	987
**LZD**	2623	426
**PMB**	1304	106
**Nisin**	2991	1293
**Untreated**	1018	633
**DPA 154**	2490	318
**DPA 156**	1715	350

Table S14. Total count of bacteria used for cytological profiling studies.

B. subtilis composite images

Ampicillin

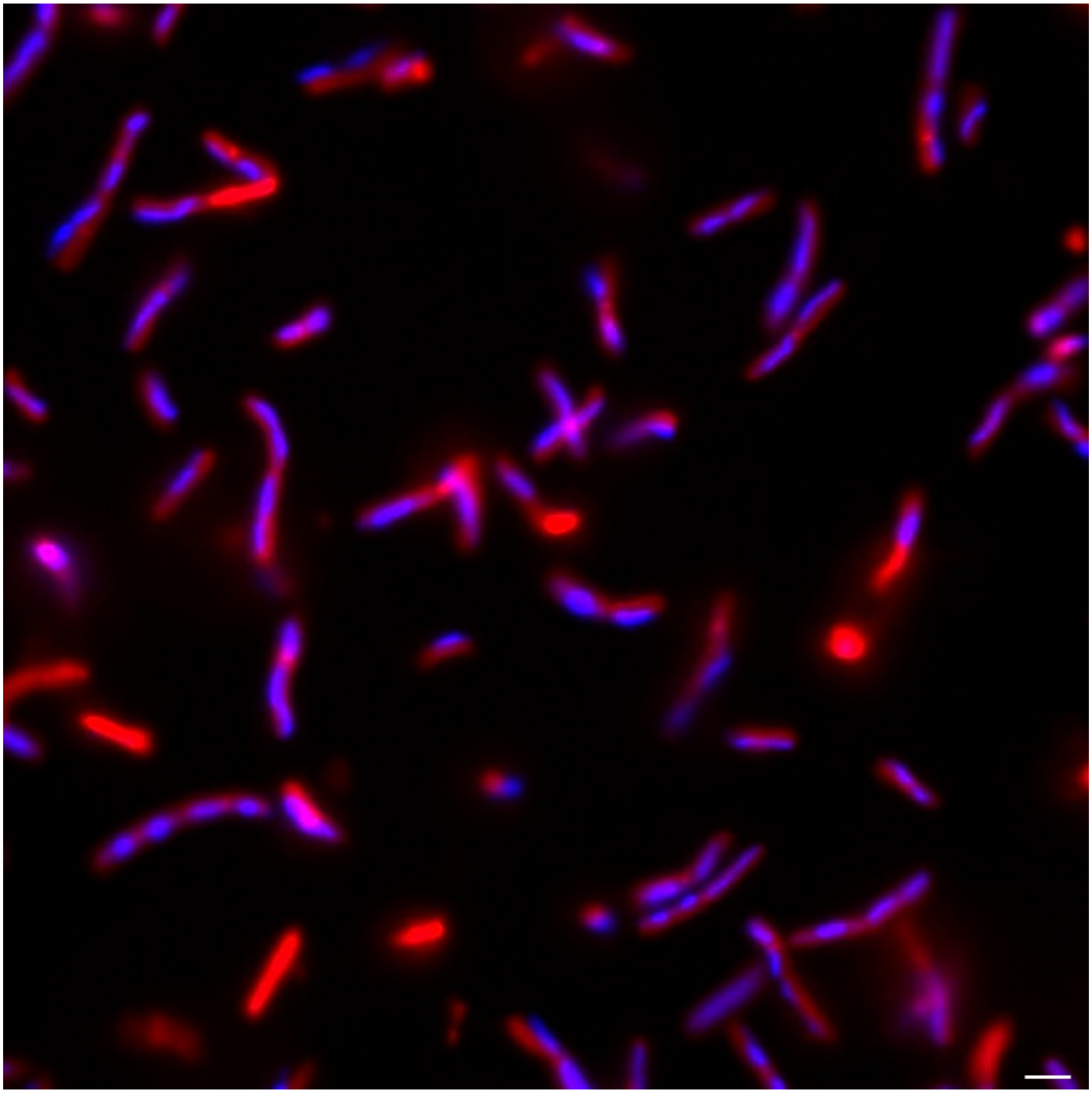

Figure S5. Cytological profile of Ampicillin-treated *B. subtilis* treated with DAPI (blue) and FM464 (Red). Corresponding analysis of expected and observed cytological profiles are discussed in the main manuscript. Scale bar 1 µM.

Vancomycin

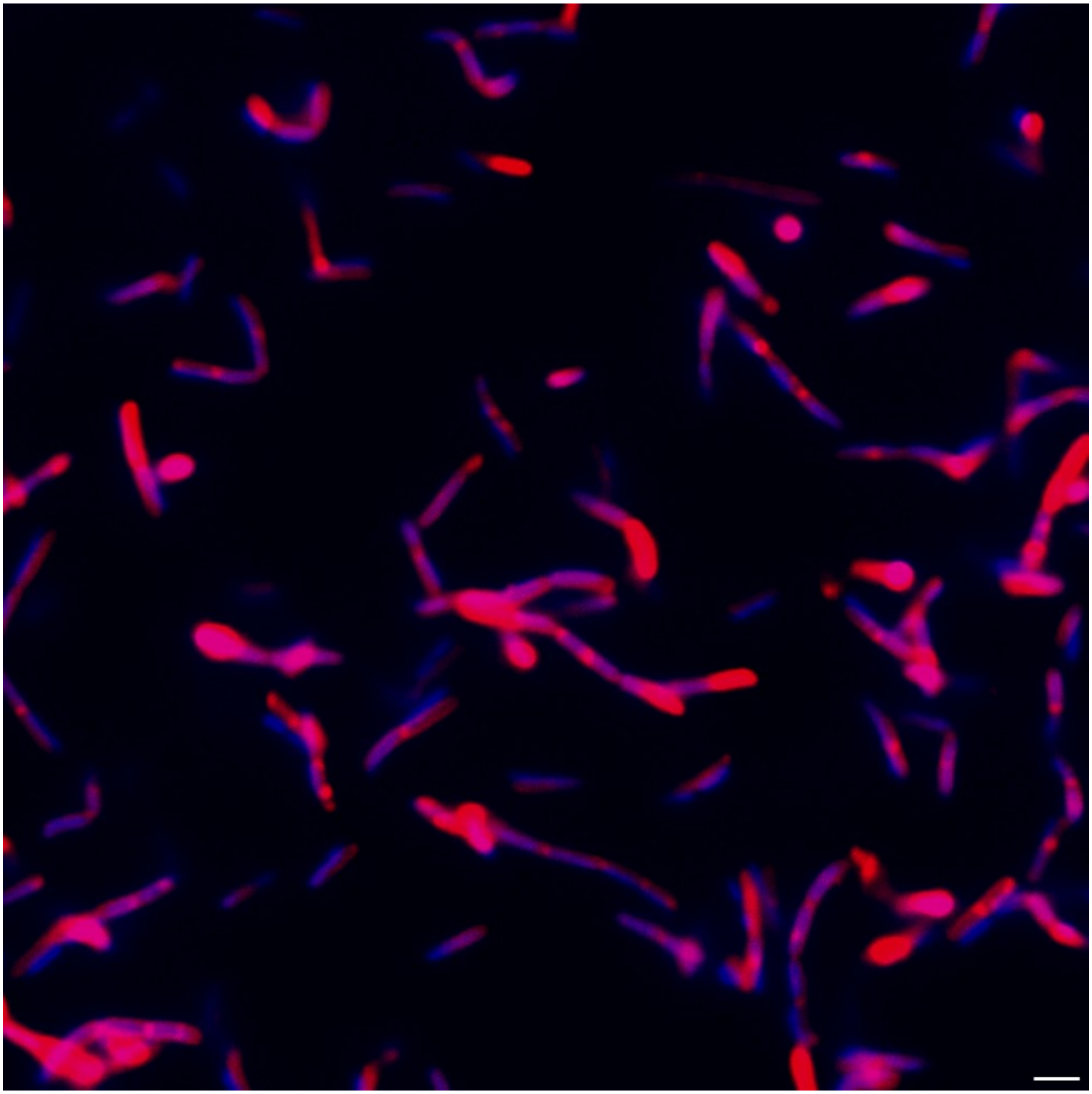

Figure S6. Cytological profile of vancomycin-treated *B. subtilis* treated with DAPI (blue) and FM464 (Red). Corresponding analysis of expected and observed cytological profiles are discussed in the main manuscript. Scale bar 1 μM.

Ciprofloxacin

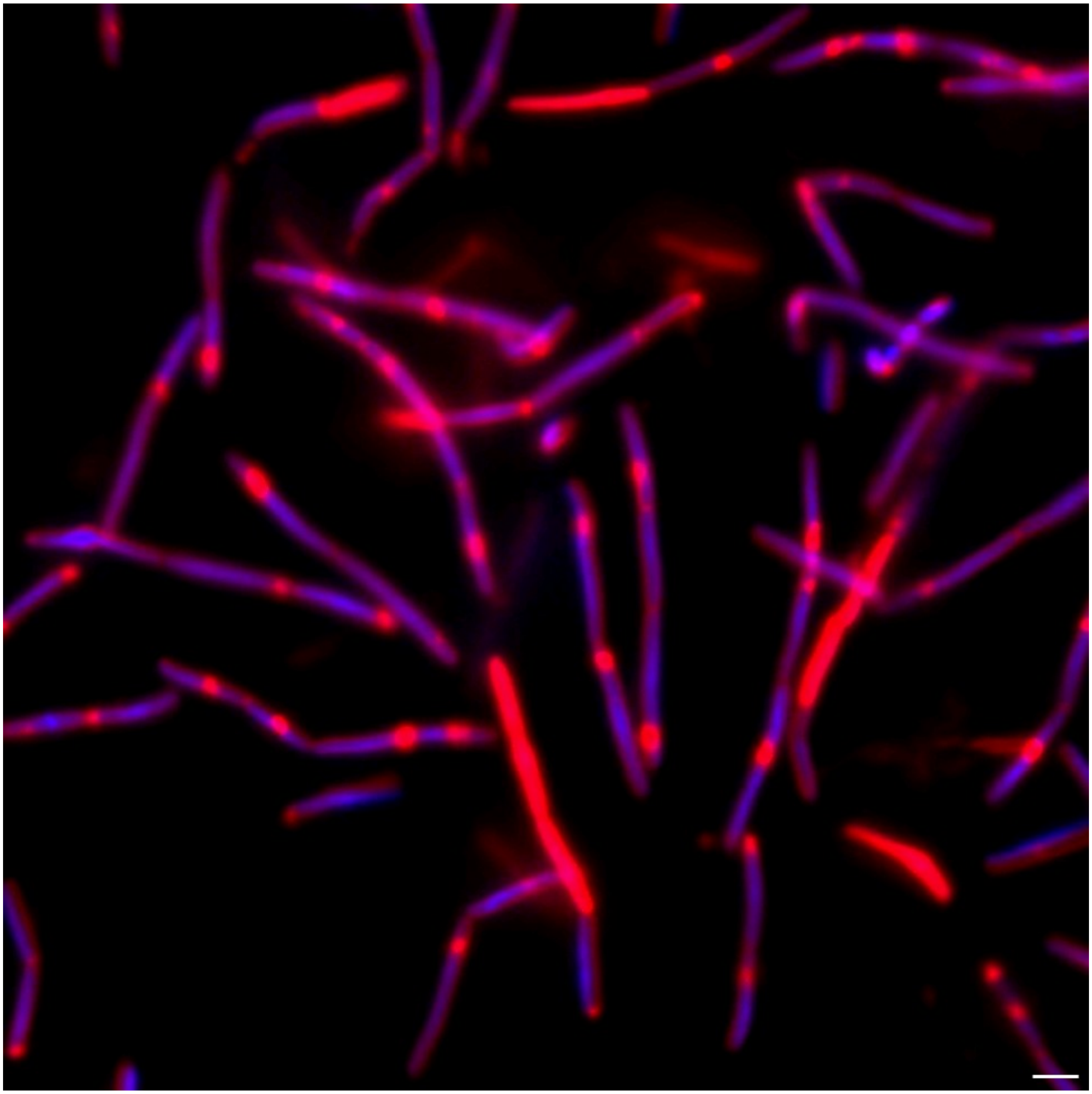

Figure S7. Cytological profile of Ciprofloxacin-treated *B. subtilis* treated with DAPI (blue) and FM464 (Red). Corresponding analysis of expected and observed cytological profiles are discussed in the main manuscript. Scale bar 1 μM.

Nalidixic acid

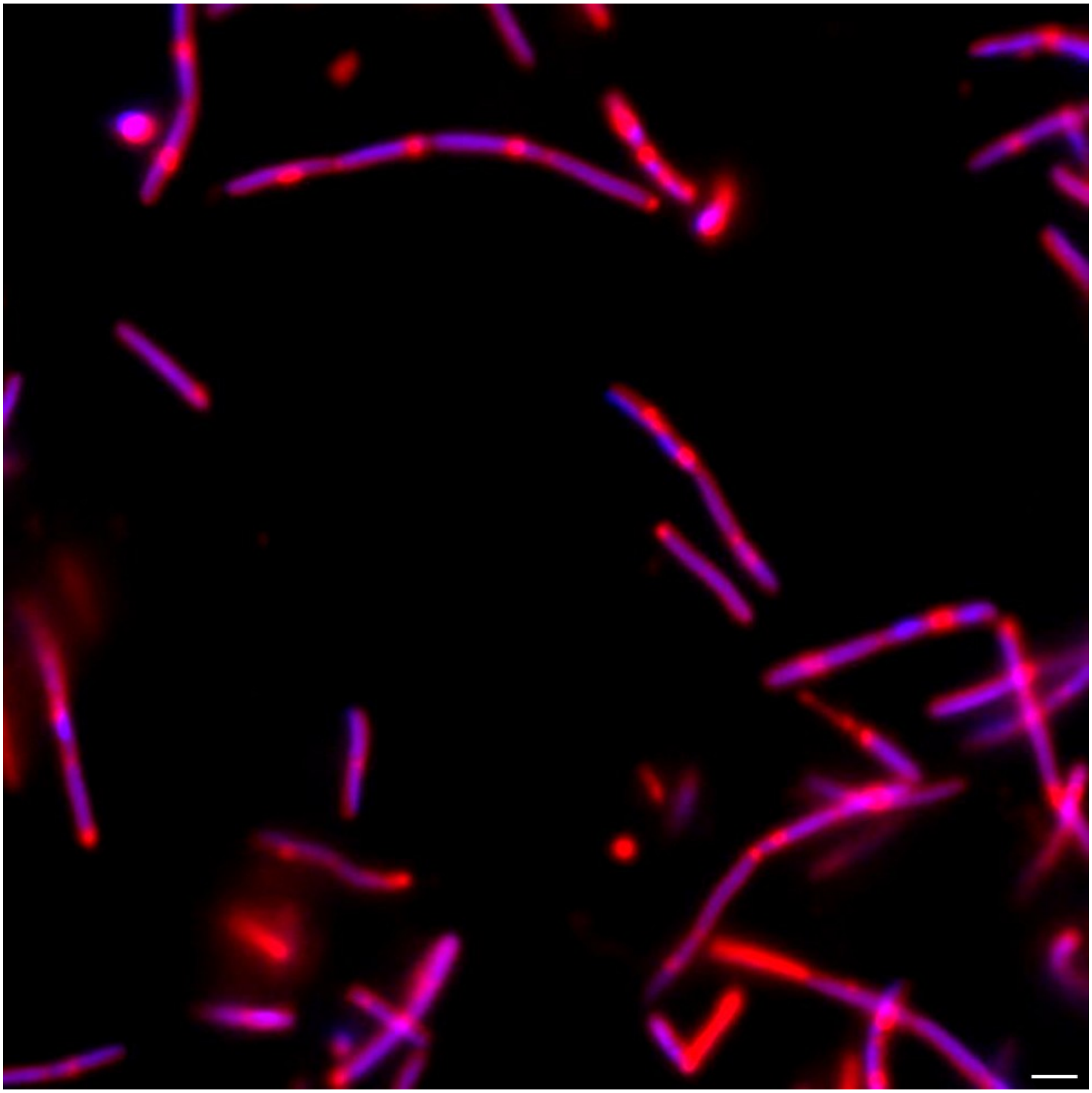

Figure S8. Cytological profile of Nalidixic acid-treated *B. subtilis* treated with DAPI (blue) and FM464 (Red). Corresponding analysis of expected and observed cytological profiles are discussed in the main manuscript. Scale bar 1 μM.

Tobramycin

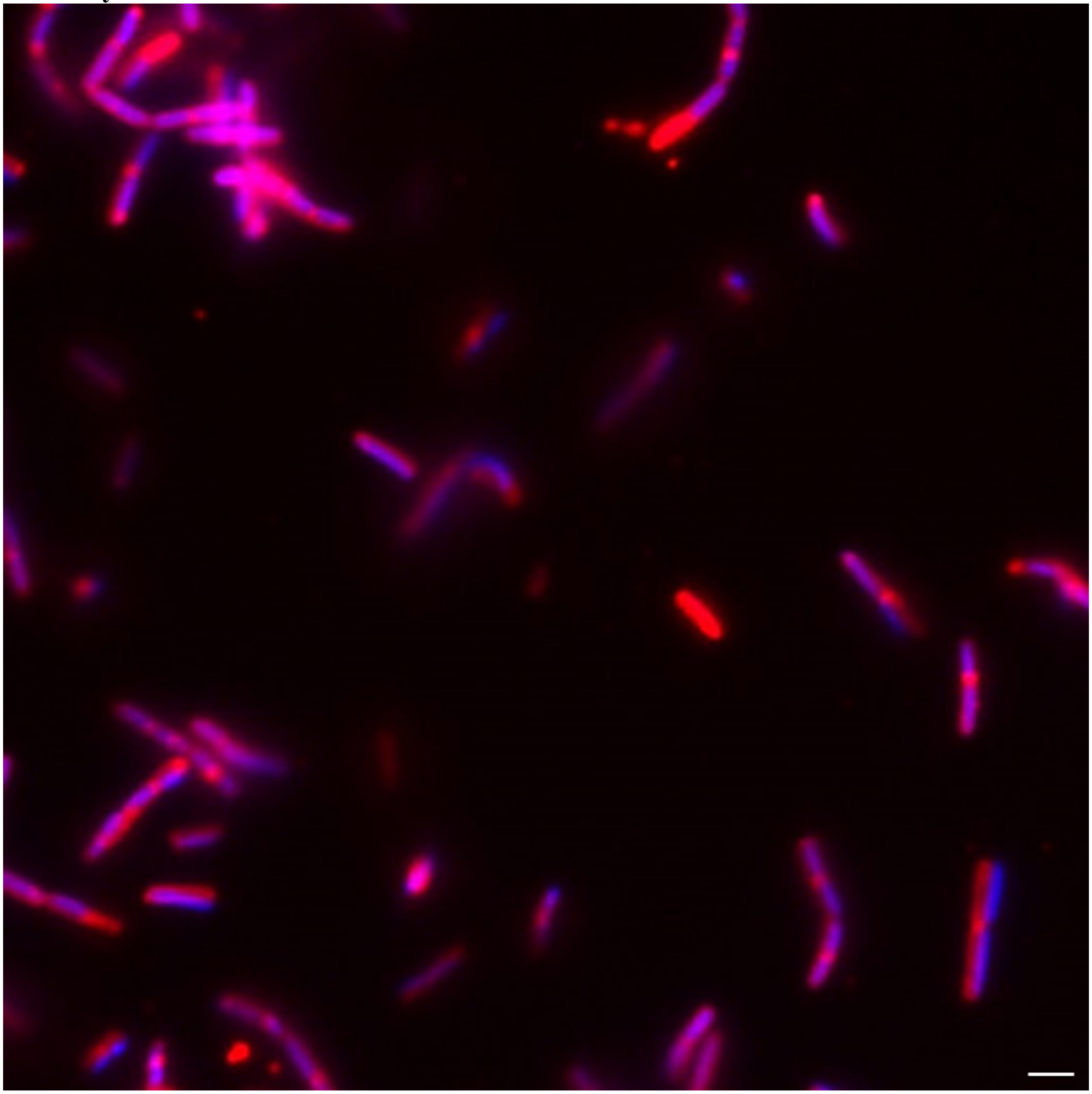

Figure S9. Cytological profile of Tobramycin-treated *B. subtilis* treated with DAPI (blue) and FM464 (Red). Corresponding analysis of expected and observed cytological profiles are discussed in the main manuscript. Scale bar 1 µM.

Neomycin

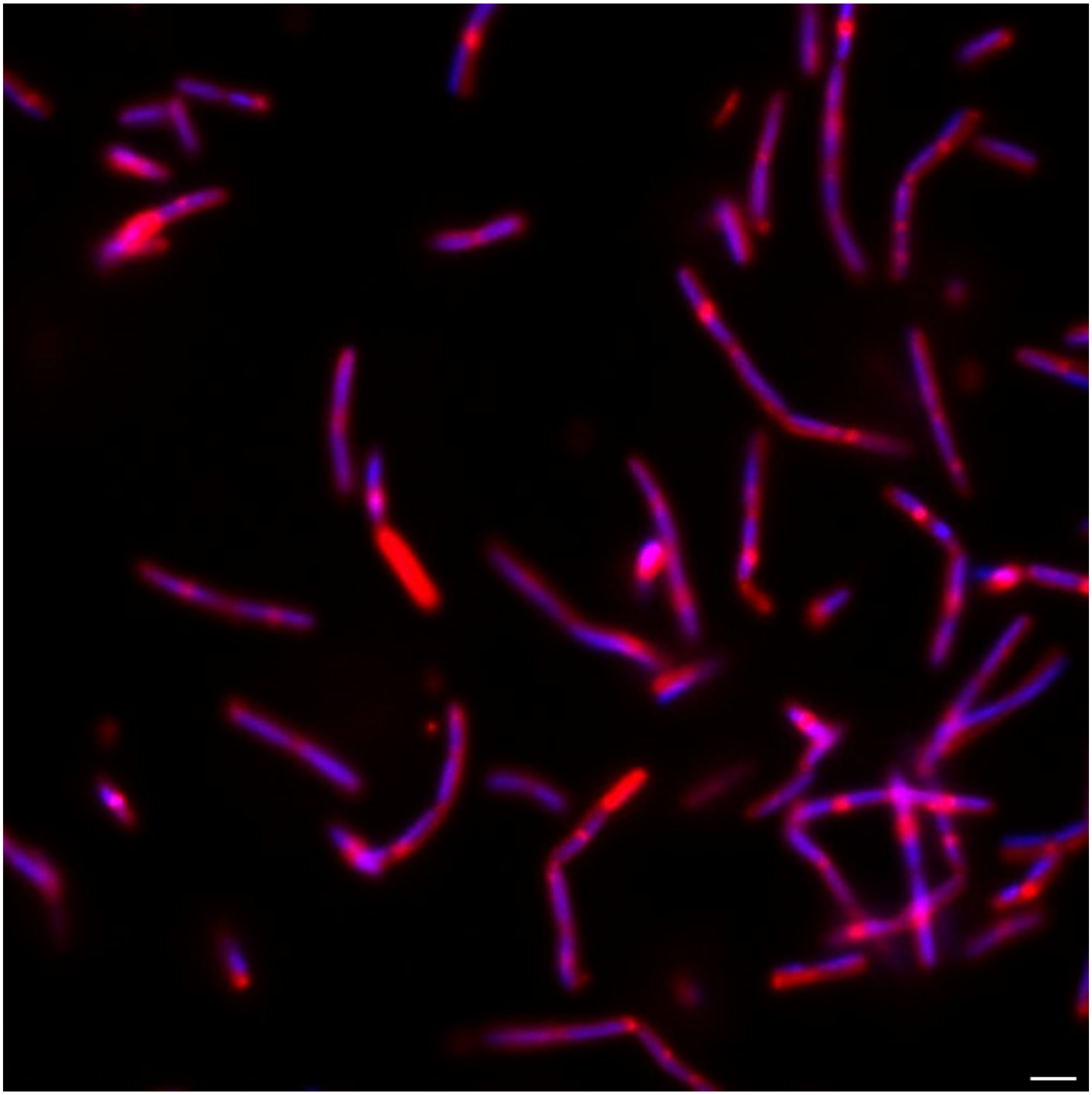

Figure S10. Cytological profile of Neomycin-treated *B. subtilis* treated with DAPI (blue) and FM464 (Red). Corresponding analysis of expected and observed cytological profiles are discussed in the main manuscript. Scale bar 1 μM.

Kanamycin

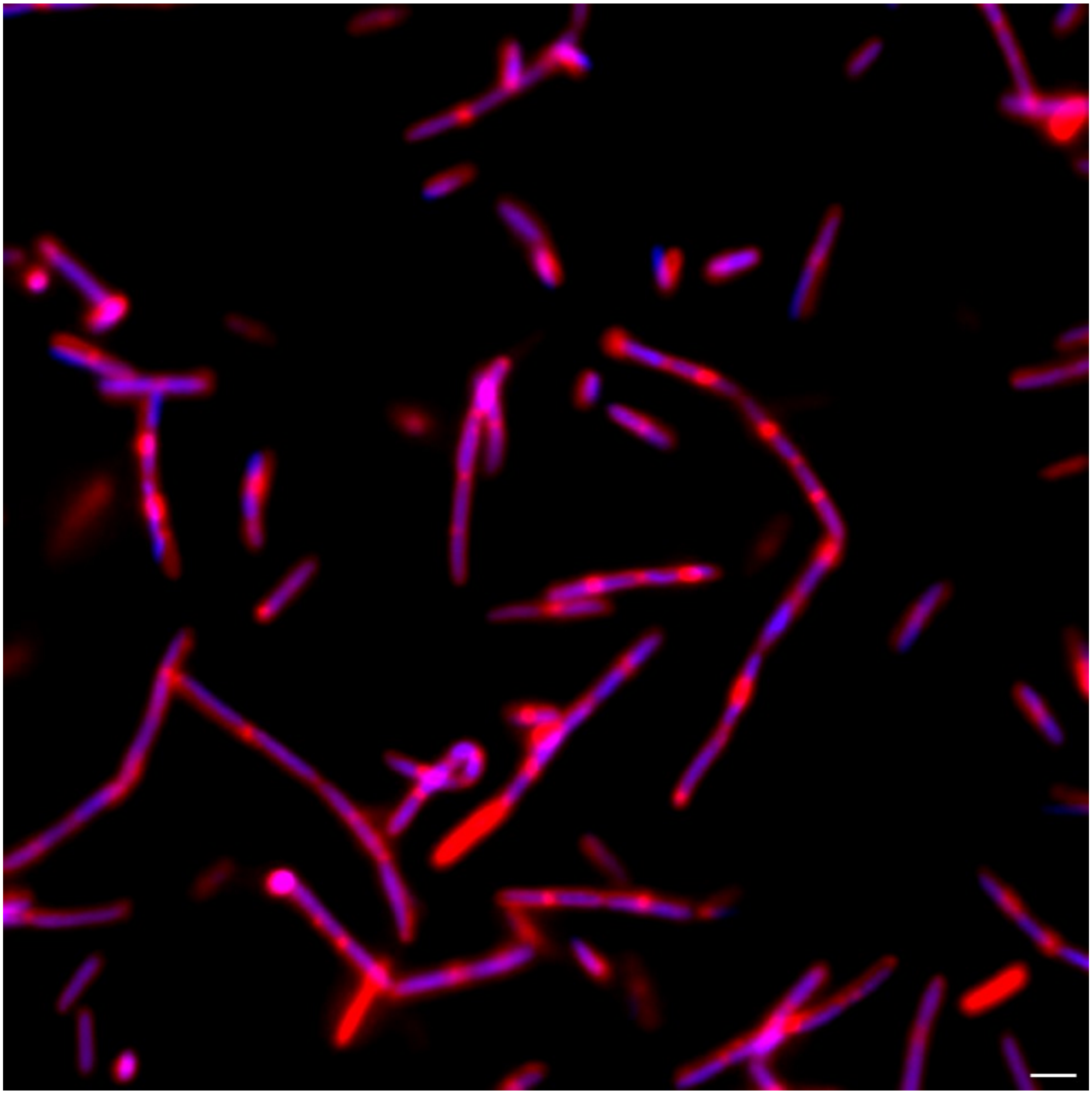

Figure S11. Cytological profile of Kanamycin-treated *B. subtilis* treated with DAPI (blue) and FM464 (Red). Corresponding analysis of expected and observed cytological profiles are discussed in the main manuscript. Scale bar 1 μM.

Linezolid

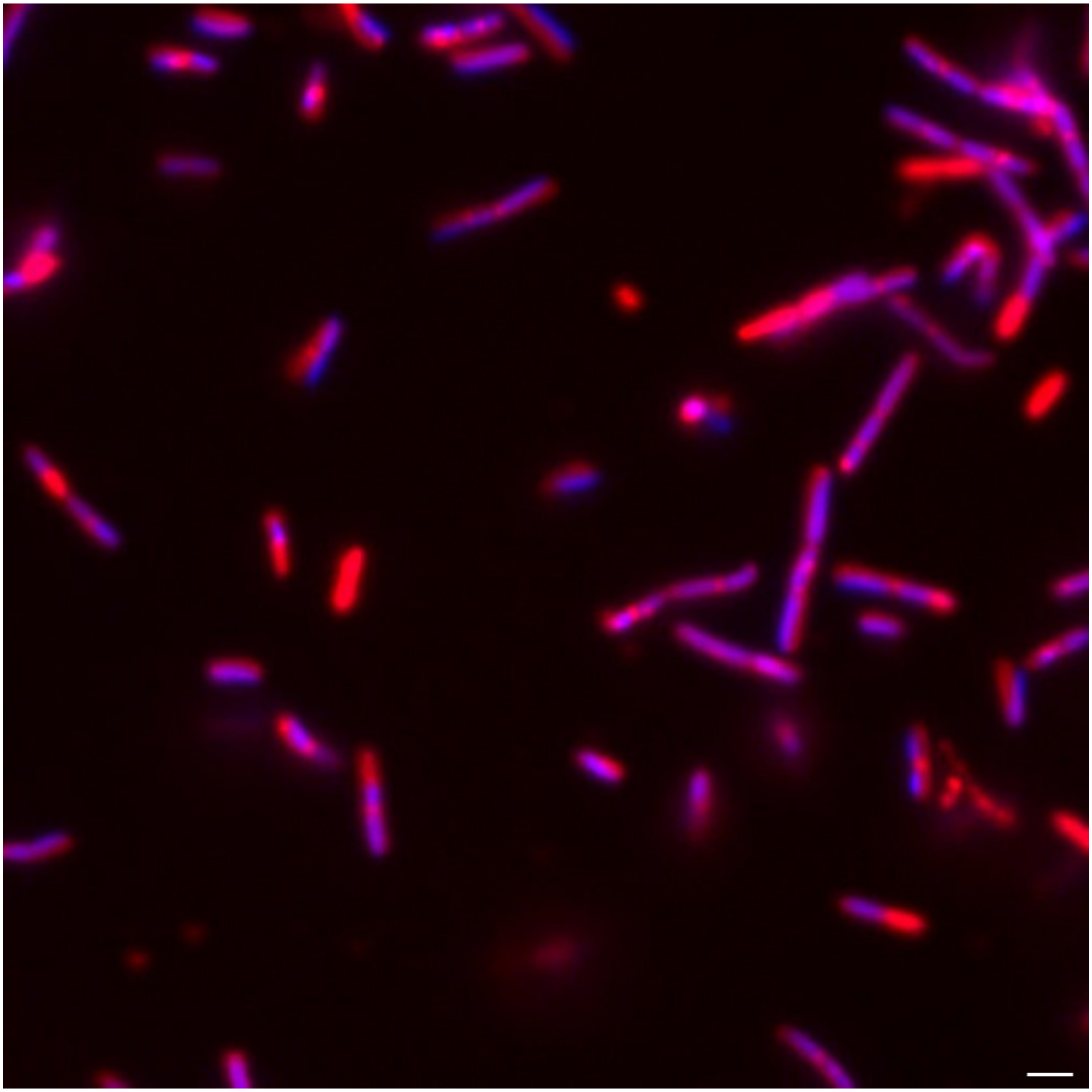

Figure S12. Cytological profile of Linezolid-treated *B. subtilis* treated with DAPI (blue) and FM464 (Red). Corresponding analysis of expected and observed cytological profiles are discussed in the main manuscript. Scale bar 1 μM.

Tetracycline

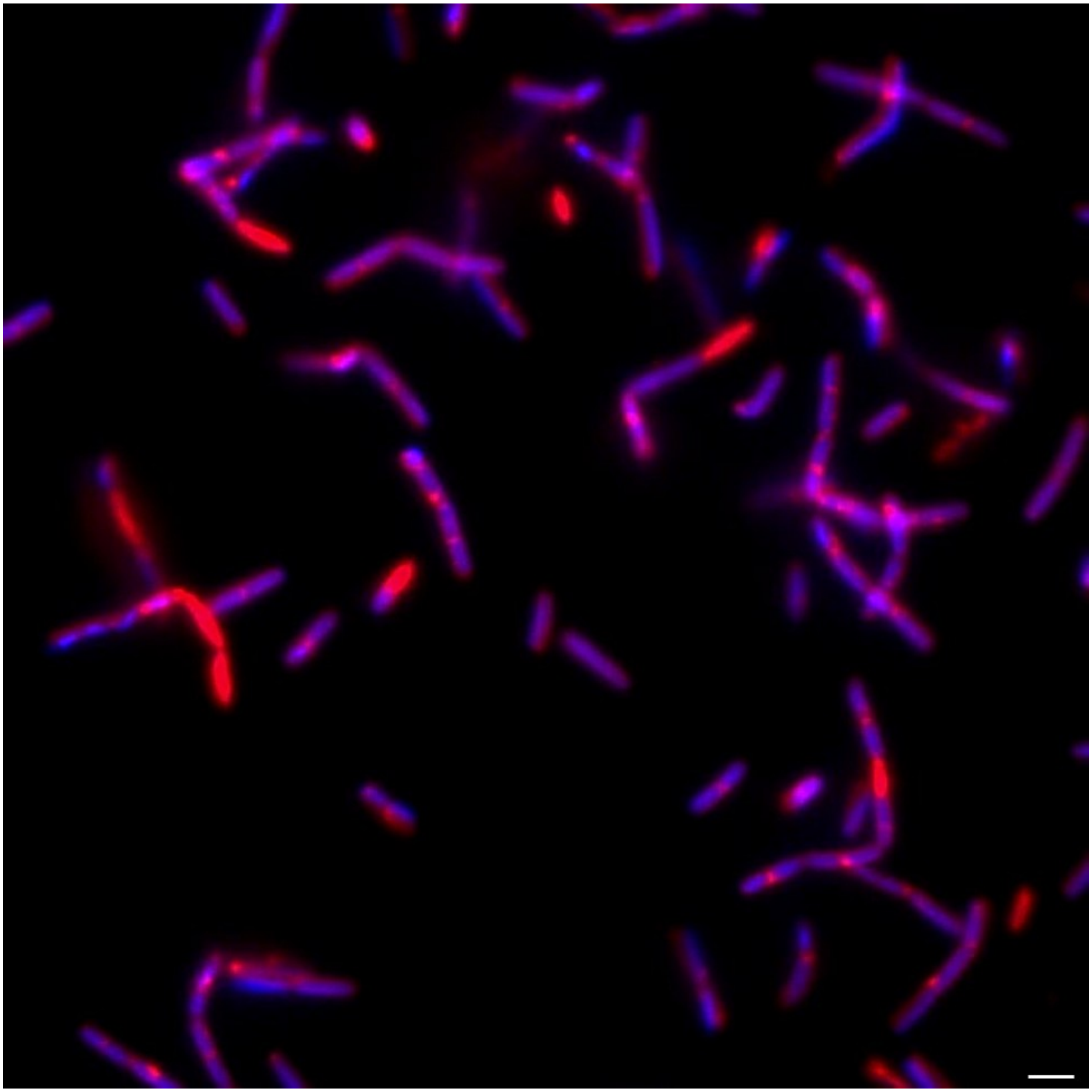

Figure S13. Cytological profile of Tetracycline-treated *B. subtilis* treated with DAPI (blue) and FM464 (Red). Corresponding analysis of expected and observed cytological profiles are discussed in the main manuscript. Scale bar 1 μM.

Rifampicin

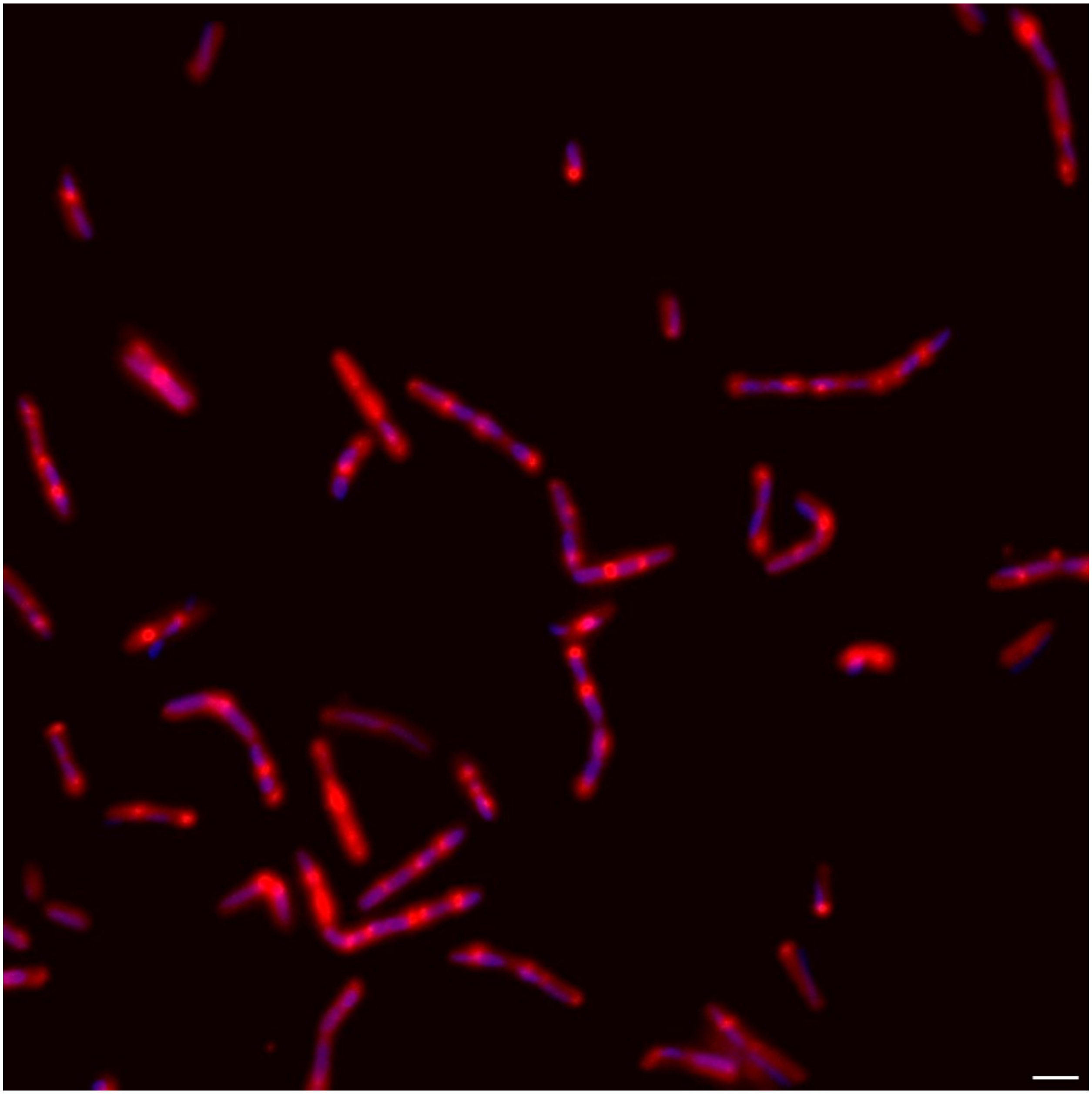

Figure S14. Cytological profile of Rifampicin-treated *B. subtilis* treated with DAPI (blue) and FM464 (Red). Corresponding analysis of expected and observed cytological profiles are discussed in the main manuscript. Scale bar 1 µM.

Nisin

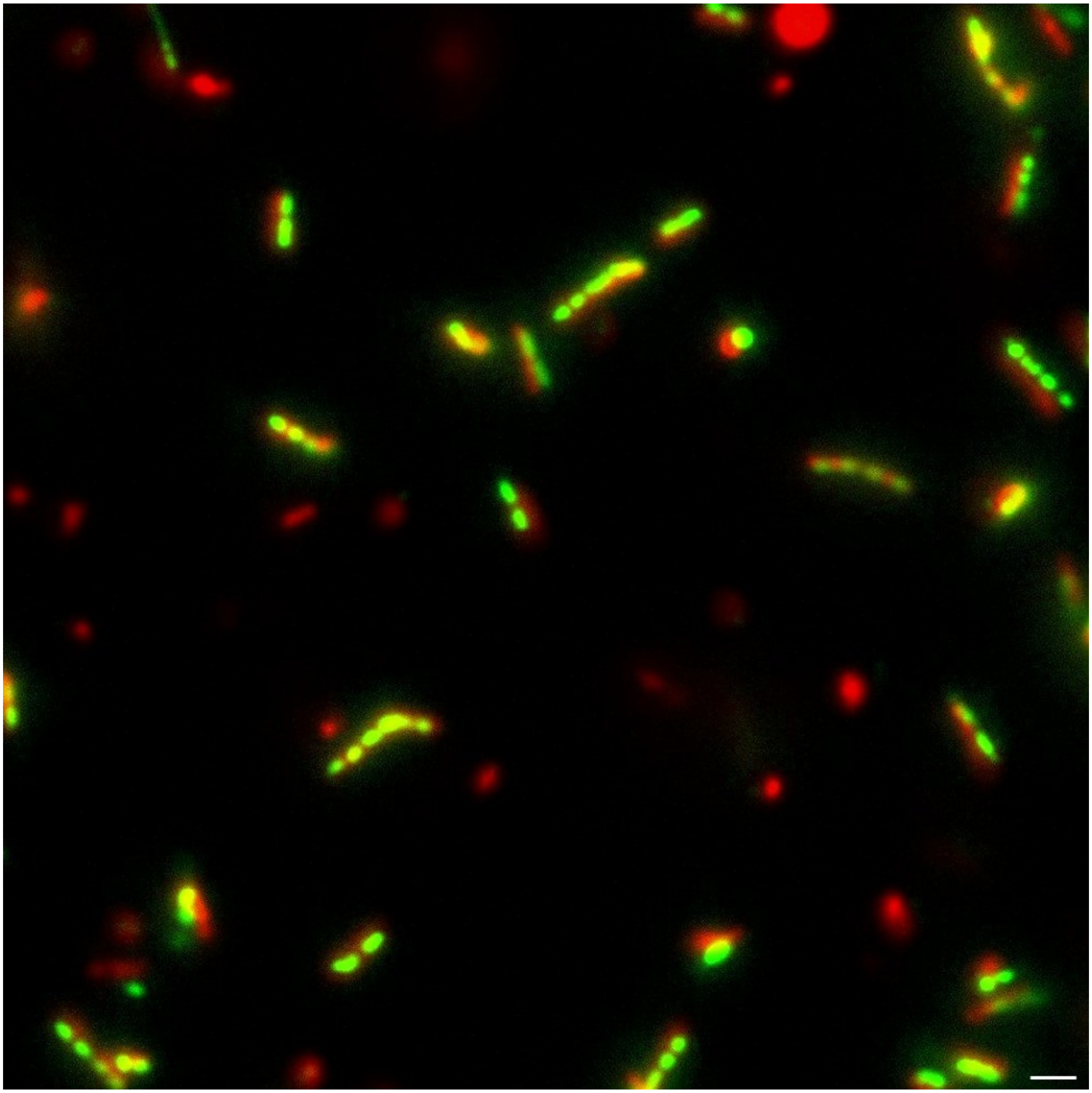

Figure S15. Cytological profile of Nisin-treated *B. subtilis* treated with SytoxG (green) and FM4-64 (red). Corresponding analysis of expected and observed cytological profiles are discussed in the main manuscript. Scale bar 1 μM.

Polymyxin B

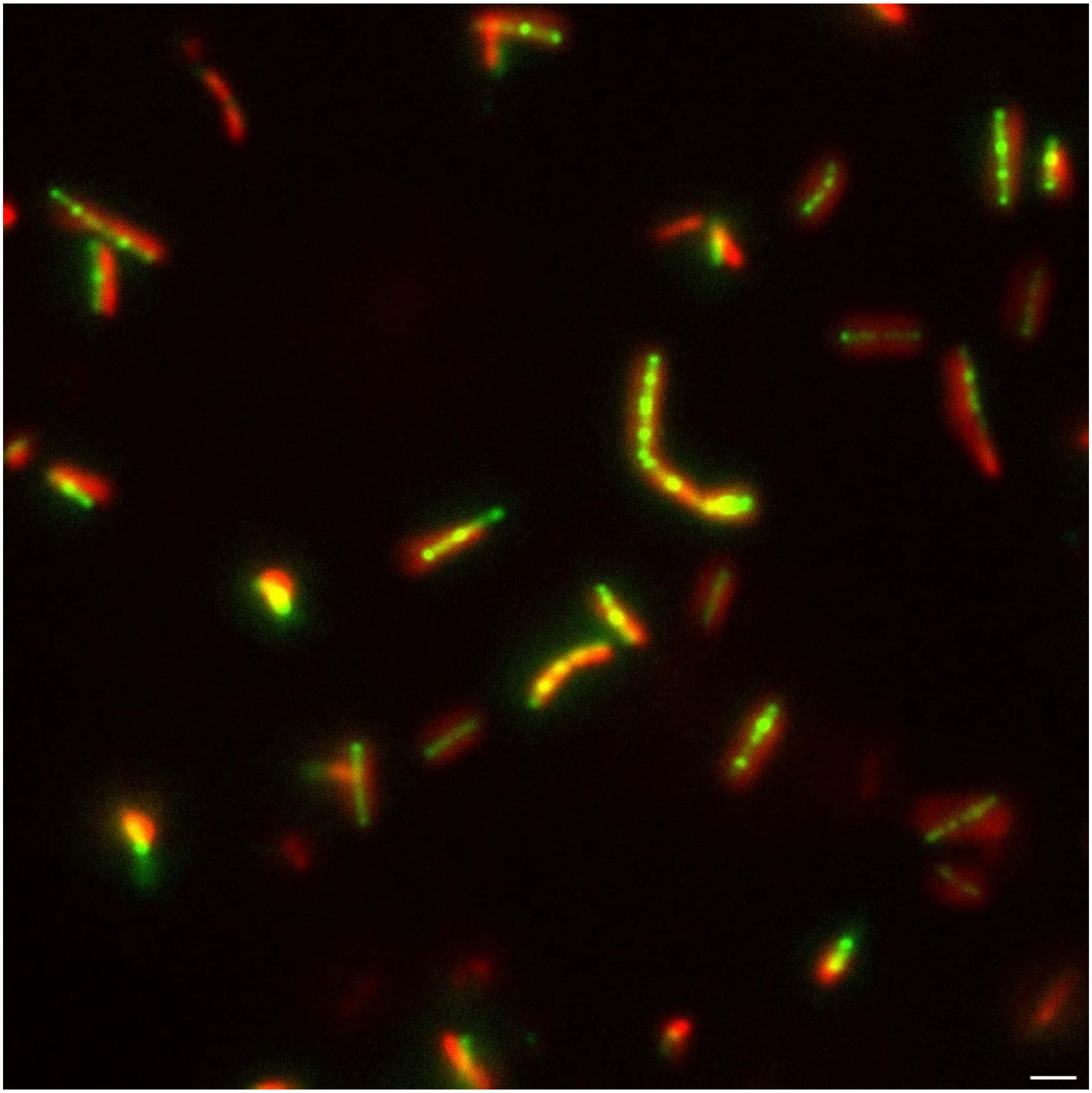

Figure S16. Cytological profile of Polymyxin-treated *B. subtilis* treated with SytoxG (green) and FM4-64 (red). Corresponding analysis of expected and observed cytological profiles are discussed in the main manuscript. Scale bar 1 μM.

DPA 154

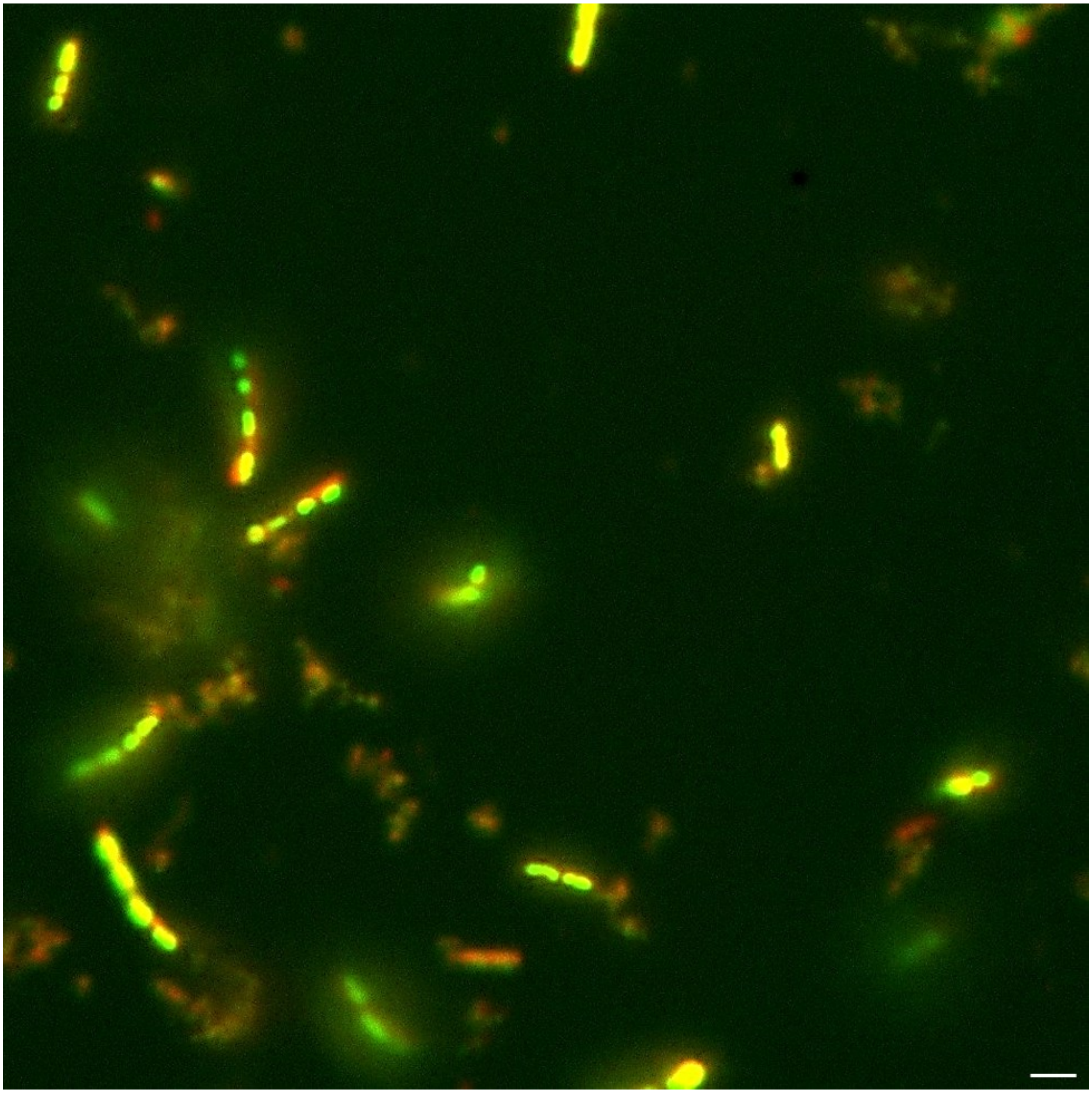

Figure S17. *B. subtilis* treated with DPA 154 and dyed with FM4-64 (red) and SytoxG (green). Observed morphologies are discussed in the manuscript. Lysed cellular contents and SytoxG infiltrates are observed in ovoid and filamentous remnant cells. Scale bar 1 µM.

Untreated

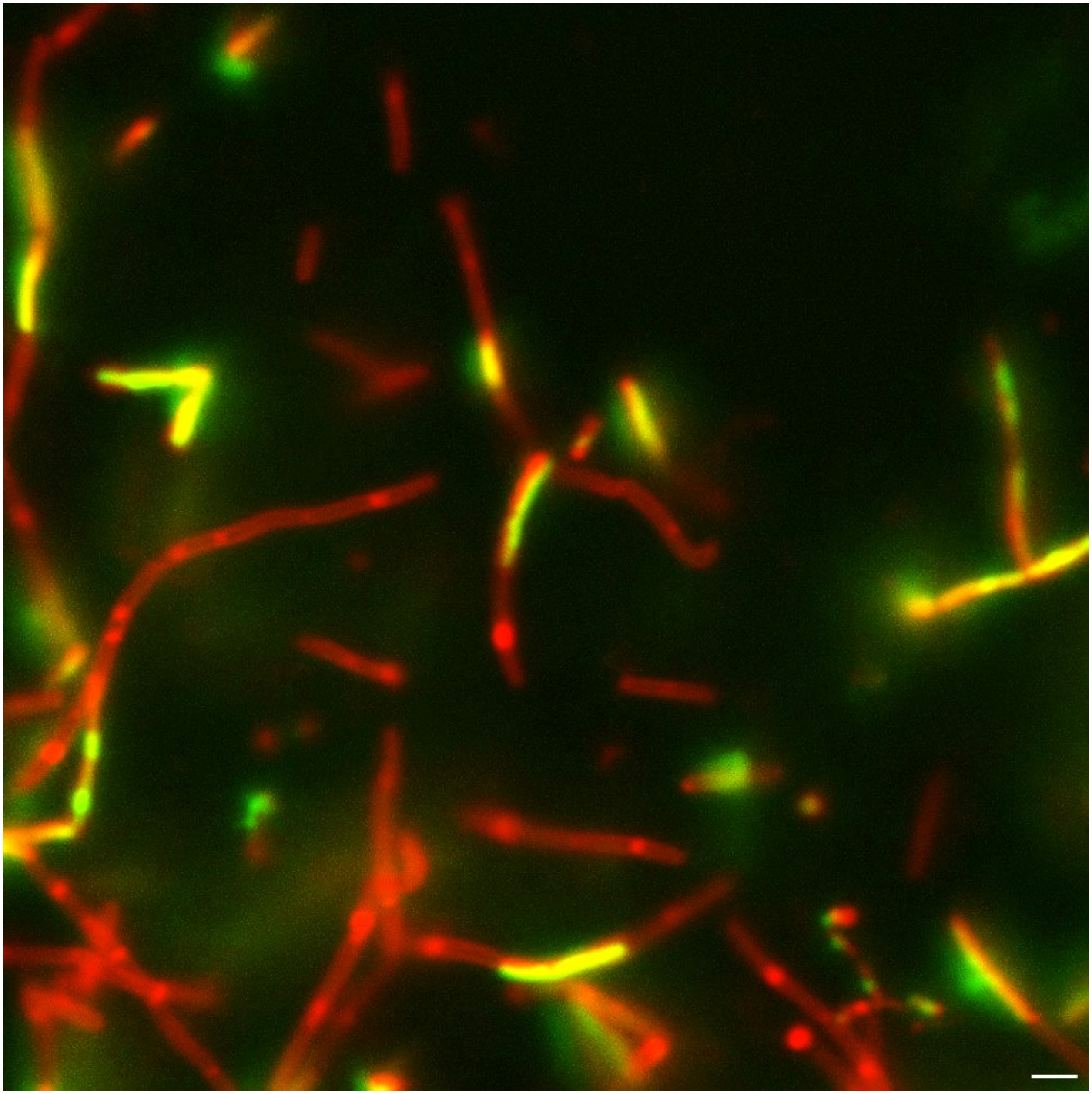

Figure S18. Cytological profile of untreated *B. subtilis* treated with SytoxG (green) and FM4-64 (red). Corresponding analysis of expected and observed cytological profiles are discussed in the main manuscript. Scale bar 1 µM.

	Ciprofloxacin vs Untreated	Ciprofloxacin vs Hoechst 156	Ciprofloxacin vs Hoechst 154	Untreated vs Hoechst 156	Untreated vs Hoechst 154	Hoechst 156 vs Hoechst 154	P
**Cell Area**	0.9952	0.7907	**0.0005**	0.5349	**<0.0000**	**0.0420**	**<0.0000**
**Cell Diameter**	0.9892	0.9980	0.4298	0.9603	0.0814	0.6131	0.1260
**Cell Form Factor**	0.2578	0.9957	**<0.0000**	0.5066	**<0.0000**	**<0.0000**	**<0.0000**
**Cell Length**	0.6897	0.9773	**<0.0000**	0.4410	**<0.0000**	**<0.0000**	**<0.0000**
**Cell Perimeter**	0.3366	0.9953	**<0.0000**	0.6033	**<0.0000**	**<0.0000**	**<0.0000**
**DNA Area**	0.5621	0.3778	0.9872	0.8696	0.2095	0.1752	0.0953
**DNA Diameter**	**<0.0000**	0.9377	0.6876	**<0.0000**	**<0.0000**	0.3499	**<0.0000**
**DNA Form Factor**	**<0.0000**	0.9608	0.0607	**<0.0000**	**<0.0000**	0.2786	**<0.0000**
**DNA Length**	**0.0006**	0.9525	0.7307	**0.0215**	**0.0111**	0.9797	**<0.0000**
**DNA Perimeter**	**0.0317**	0.5232	0.7608	0.8407	0.2500	0.9446	**0.0296**

Table S15. One-way ANOVA test comparing the means of each of the chosen cell morphology measurements evaluated against each individual treatment. $\alpha = 0.05$. Significant results are labeled in bold. Individual treatment P-values were calculated using a Tukey HSD Post-Hoc test for every observation. DPA 154, but not 156, shows significant morphology differences from ciprofloxacin.

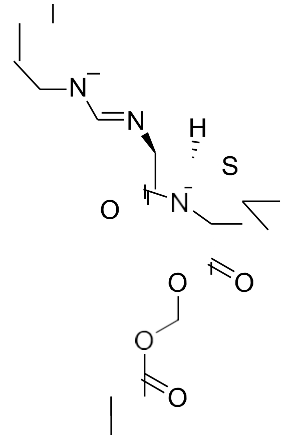

Pivmecillinam
Cell Wall Synthesis

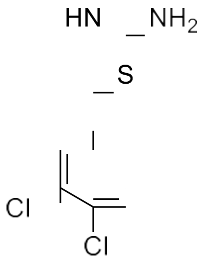

A22 [S-(3,4-Dichlorobenzyl)isothiourea]
Cytoskeleton Inhibitor

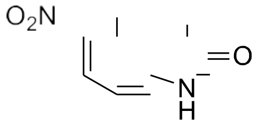

5-nitro-2-oxyindole
Efflux Pump Inhibitor

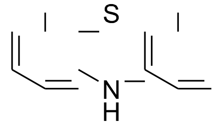

Phenothiazine
Efflux Pump Inhibitor

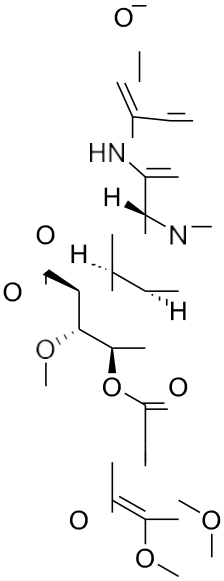

Reserpine
Efflux Pump Inhibitor

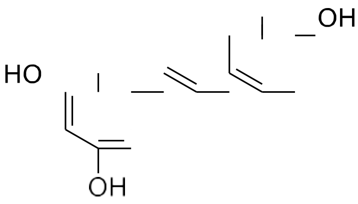

Resveratrol
Efflux Pump Inhibitor

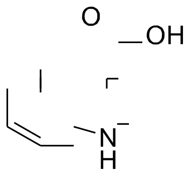

Indole-3-carboxylic acid
Efflux Pump Inhibitor

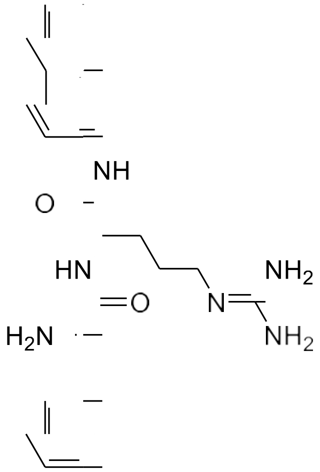

Phenylalanine-Arginine Beta-Napthylamide
Efflux Pump Inhibitor

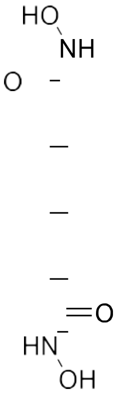

Suberohydroxamic acid
Efflux Pump Inhibitor

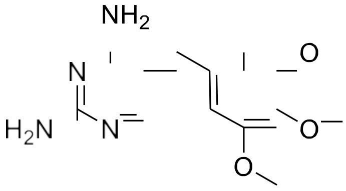

Trimethoprim
Folic Acid Synthesis

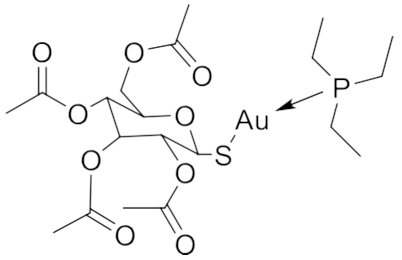

Auranofin
Multiple Targets

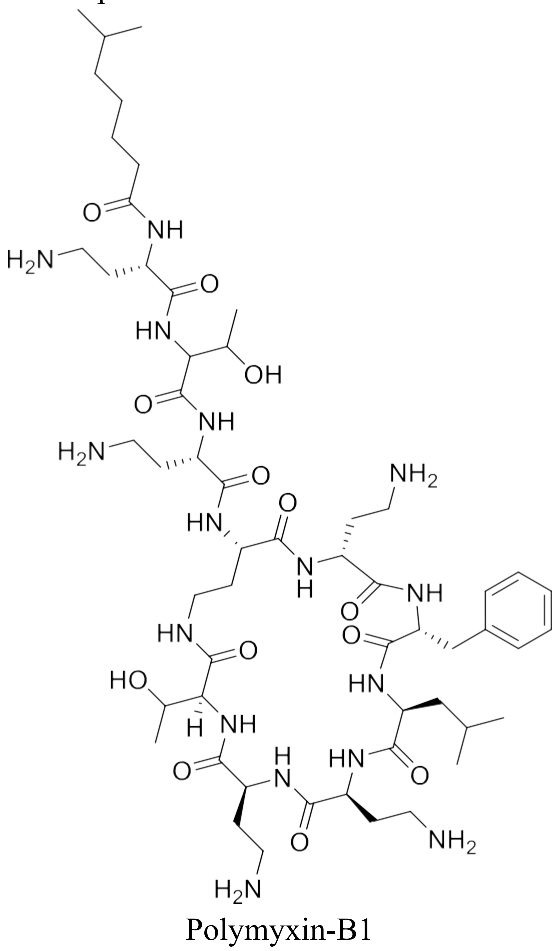

Polymyxin-B1
Outer Membrane Active

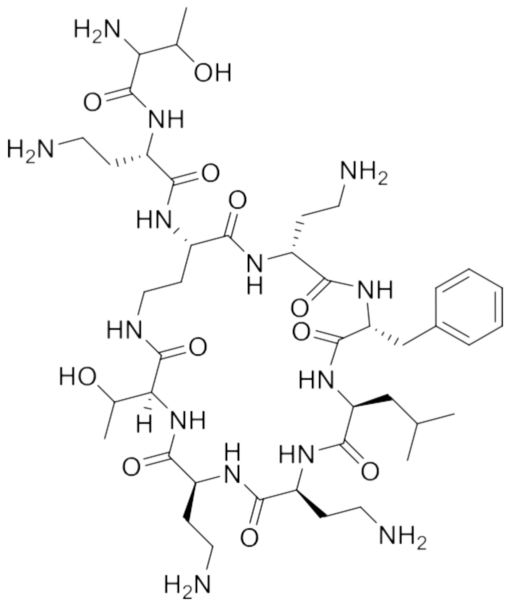

Polymyxin-B Nonapeptide
Outer Membrane Active

Figure S19. Structures of all synergy compounds used in this study.

Table S16. Concentrations of test compounds used in synergy assays and minimal inhibitory concentration in bacteria.

			MIC (µM)			
**Compounds**	**Conc. (µM)**	**Mechanism**	***E. coli*** **25922**	***E. coli*** **H4H**	***K. pneumoniae*** **NR15410**	***P. aeruginosa*** **27853**
Pivmecillinam[14]	10	Cell Wall	12.5	25	>50	>50
A22 [15]	50	Cytoskeleton	>50	>50	>50	>50
5-nitro-2-oxylndole[16]	100	Efflux Pump	>200	>200	>200	>200
Phenothiazine[17]	100	Efflux Pump	>200	>200	>200	>200
Reserpine[18]	100	Efflux Pump	>200	>200	>200	>200
Reservatrol[19]	100	Efflux Pump	>200	>200	>200	>200
Indole-3-carboxylic acid[20]	100	Efflux Pump	>200	>200	>200	>200
Phe-Argβ-napthylamide[21]	100	Efflux Pump	>200	>200	>200	>200
Trimethoprim[22]	100	Folic Acid	>32	>32	>50	>200
Suberohydroxamic acid[23]	100	Iron Chelation	>200	>200	>200	>200
Polymyxin-B[24]	0.39	Outer Membrane	1.25	2	6.25	12.5
Polymyxin-B Nonapeptide[25]	100	Outer Membrane	>200	>200	>200	>200
Auranofin[26]	20	Multiple Targets	>64	>32	>50	>200

Abbreviations used: Pivmecillinam (Piv), A22 (S-(3,4-Dichlorobenzyl)isothiourea), 5-nitro-2-oxylndole (Nitro), Phenothiazine (Pheno), Reserpine (Reserp), Resveratrol (Resver), Indole-3-carboxylic acid (I3C), Phe-Argβ-naphtylamide (PaβN), Trimethoprim (Tri), Suberohydroxamid acid (Subero), Polymyxin-B (PMB), polymyxin-B nonapeptide (PMBN), Auranofin (Aur).

Checkerboard synergy assays of *ESKAPE* Pathogens

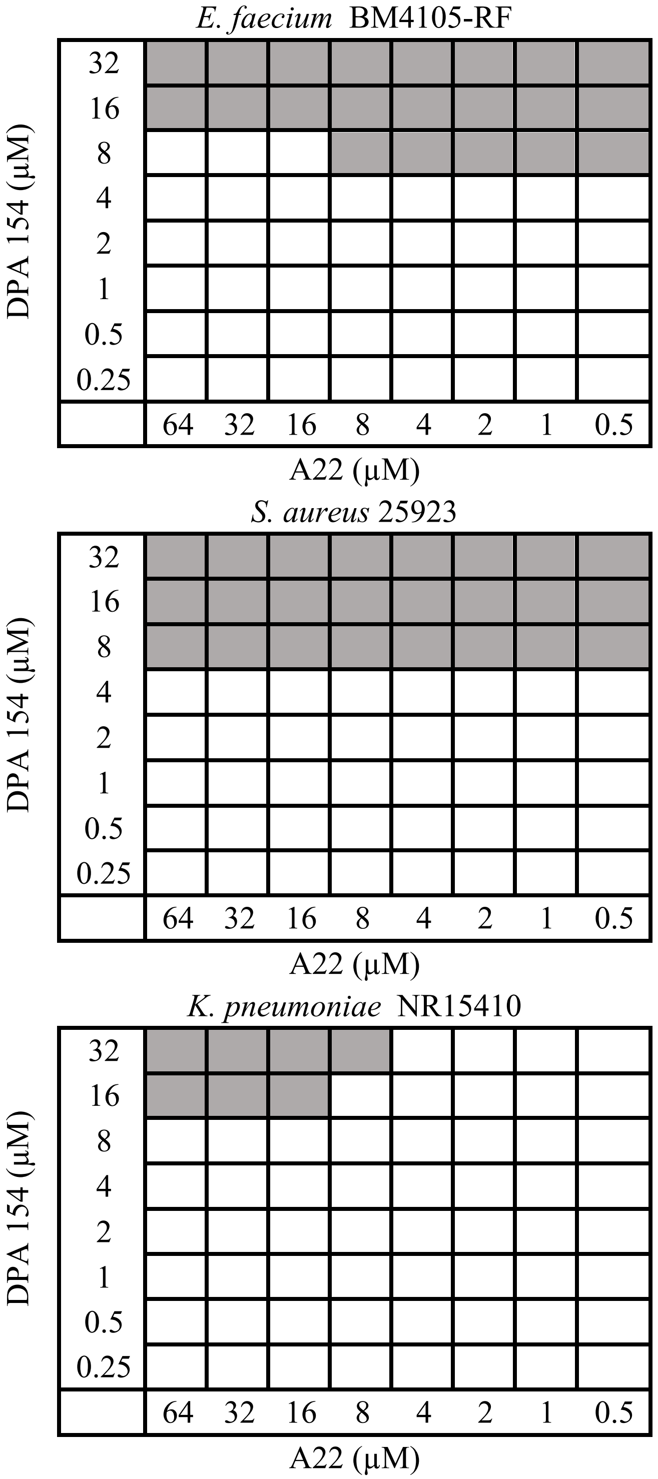

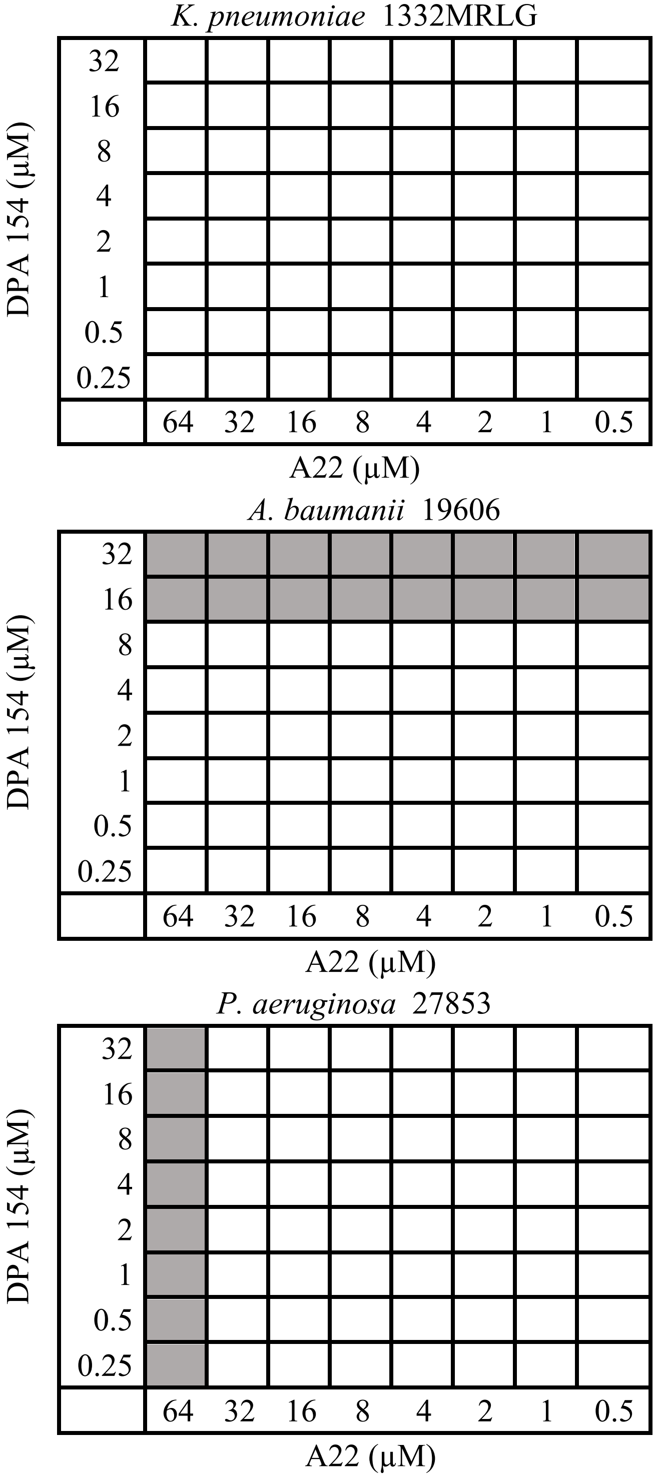
K. pneumoniae 1332MRLG
DPA 154 (µM)
32 16 8 4 2 1 0.5 0.25
64 32 16 8 4 2 1 0.5
A22 (µM)
A. baumanii 19606
DPA 154 (µM)
32 16 8 4 2 1 0.5 0.25
64 32 16 8 4 2 1 0.5
A22 (µM)
P. aeruginosa 27853
DPA 154 (µM)
32 16 8 4 2 1 0.5 0.25
64 32 16 8 4 2 1 0.5
A22 (µM)

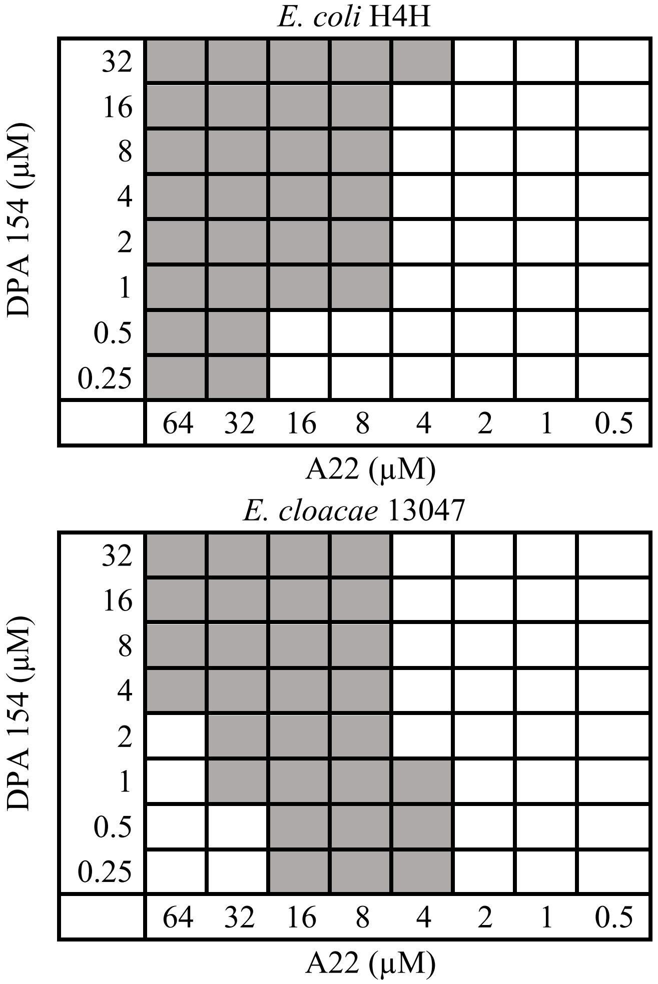

Figure S20. Representations of checkerboard synergy assays of DPA 154 and A22 evaluation for synergy across all *ESKAPE* pathogens. Synergy is observed as stepwise inhibition and quantified by FIC index calculation.

	MIC of DPA 154 (µM) in combination with antibiotic			
**Compound(s)**	***E. coli*** **29522**	***E. coli*** **H4H**	***K. Pneumoniae*** **NR15410**	***P. Aeruginosa*** **27853**
Hoechst 33258	30^{α}	32	35^{α}	30^{α}
Hoechst 33342	18^{α}	4	15^{α}	18^{α}
DPA 153	>64	>64	>64	>64
DPA 154	64	32	>64	>64
DPA 156	>64	>64	>64	>64
Hoechst 33258 + PolyB	16	4-8	4	8
Hoechst 33342 + PolyB	4-8	1-2	2	4-8
DPA 153 + PolyB	16	4	16	8
DPA 154 + PolyB	16	8	8	16
DPA 156 + PolyB	16-32	4-8	16-32	16-32
Hoechst 33258 + PMBN	>32	>32	>32	>32
Hoechst 33342 + PMBN	2	2	2	1
DPA 153 + PMBN	>32	>32	>32	>32
DPA 154 + PMBN	16	16-32	16	32
DPA 156 + PMBN	>32	>32	>32	>32
Hoechst 33258 + PAβN	>32	>32	>32	>32
Hoechst 33342 + PAβN	0.5	0.5	2	2
DPA 153 + PAβN	>32	>32	>32	>32
DPA 154 + PAβN	2-4	2-4	32	16
DPA 156 + PAβN	>32	>32	>32	>32
Hoechst 33258 + Tri	>32	>32	>32	>32
Hoechst 33342 + Tri	32	8-16	16-32	>32
DPA 153 + Tri	>32	>32	>32	>32
DPA 154 + Tri	32	32	>32	>32
DPA 156 + Tri	>32	>32	>32	>32
Hoechst 33258 + Aur	>32	>32	>32	>32
Hoechst 33342 + Aur	8-16	8-16	16-32	>32
DPA 153 + Aur	>32	>32	>32	>32
DPA 154 + Aur	>32	>32	>32	>32
DPA 156 + Aur	>32	>32	>32	>32
Hoechst 33258 + A22	>32	>32	0.25	0.25
Hoechst 33342 + A22	>32	8	0.25	0.25
DPA 153 + A22	16	8	0.25	0.25
DPA 154 + A22	16	1	0.25	0.25
DPA 156 + A22	16	8	0.25	0.25
Hoechst 33258 + I3C	0.25	>32	>32	---
Hoechst 33342 + I3C	0.25	>32	>32	---
DPA 153 + I3C	0.25	>32	>32	---
DPA 154 + I3C	0.25	>32	>32	---
DPA 156 + I3C	0.25	>32	>32	---
Hoechst 33258 + Nitro	>32	>32	>32	>32
Hoechst 33342 + Nitro	>32	>32	>32	>32
DPA 153 + Nitro	>32	>32	>32	>32

DPA 154 + Nitro	>32	>32	>32	>32
DPA 156 + Nitro	>32	>32	>32	>32
Hoechst 33258 + Pheno	>32	>32	>32	>32
Hoechst 33342 + Pheno	>32	>32	>32	>32
DPA 153 + Pheno	>32	>32	>32	>32
DPA 154 + Pheno	>32	>32	>32	>32
DPA 156 + Pheno	>32	>32	>32	>32
Hoechst 33258 + Piv	>32	0.25	>32	>32
Hoechst 33342 + Piv	>32	0.25	>32	>32
DPA 153 + Piv	>32	0.25	>32	>32
DPA 154 + Piv	>32	0.25	>32	>32
DPA 156 + Piv	>32	0.25	>32	>32
Hoechst 33258 + Reserp	>32	0.25	>32	>32
Hoechst 33342 + Reserp	>32	0.25	>32	>32
DPA 153 + Reserp	>32	0.25	>32	>32
DPA 154 + Reserp	>32	0.25	>32	>32
DPA 156 + Reserp	>32	0.25	>32	>32
Hoechst 33258 + Resver	>32	>32	>32	>32
Hoechst 33342 + Resver	0.5	>32	>32	>32
DPA 153 + Resver	>32	>32	>32	>32
DPA 154 + Resver	1	>32	>32	>32
DPA 156 + Resver	16	>32	>32	>32
Hoechst 33258 + Resver	>32	>32	>32	>32
Hoechst 33342 + Resver	>32	>32	>32	>32
DPA 153 + Resver	>32	>32	>32	>32
DPA 154 + Resver	>32	>32	>32	>32
DPA 156 + Resver	>32	>32	>32	>32

Table S17. Total SPSA results for all compounds screened in this study. Compound structures found in Figure 9. $^{\alpha}$ MIC values referenced from Ranjan et al. 2017[27, 28]

	64	67	97	96	97	97	97	98
	32	44	77	98	99	96	96	99
	16	43	59	73	99	99	98	99
	8	38	53	62	93	99	99	100
A22	4	22	22	39	90	95	96	96
	2	6	19	14	47	73	97	99
	1	-2	6	5	24	36	99	99
	0.5	-3	4	19	28	42	100	95
	0	0	7	5	21	49	96	99
		0	1	2	4	8	16	32
					DPA 154			

	64	76	85	84	86	86	85	93
	32	79	52	68	79	70	81	97
	16	76	51	57	68	75	76	93
	8	60	62	70	77	77	71	91
A22	4	43	38	43	49	57	63	94
	2	21	28	31	32	51	72	97
	1	6	3	2	24	52	68	98
	0.5	-8	4	19	28	42	73	95
	0	0	-5	-2	5	43	70	92
		0	1	2	4	8	16	32
					DPA 156			

	64	67	83	88	88	88	90	90
	32	68	76	84	94	96	96	94
	16	66	25	49	60	72	78	68
	8	61	23	31	56	52	51	59
A22	4	22	24	24	28	29	29	38
	2	-3	-3	-4	9	21	30	34
	1	6	-2	1	7	7	15	33
	0.5	5	1	4	-3	3	18	37
	0	0	6	-5	-1	9	16	39
		0	1	2	4	8	16	32
					H33258			

	64	73	95	92	93	93	94	98
	32	61	86	95	91	94	91	99
	16	64	71	74	86	86	96	100
	8	53	76	78	80	82	82	100
A22	4	23	42	43	41	47	62	90
	2	4	37	43	41	47	62	81
	1	5	26	29	32	36	57	69
	0.5	4	22	27	28	42	54	73
	0	0	13	18	20	25	45	65
		0	1	2	4	8	16	32
					Ciprofloxacin			

Figure S21. Synergistic action of A22 against ciprofloxacin-resistant *Acinetobacter baumannii*.

Checkerboard broth microdilution assays between A22 and top to bottom: DPA 154, DPA 156,

H33258 and ciprofloxacin. Dark regions represent higher cell density and numbers are the percent growth inhibition as determined by optical density at A_{600nm}. The A22 / DPA 154 and A22 / ciprofloxacin combinations are synergistic by FICI calculation while synergy is not observed with A22, 156, or H33258.

A22							
64	93	97	96	97	97	97	98
32	87	82	85	98	99	100	100
16	76	74	82	95	98	100	100
8	55	59	84	96	94	95	98
4	32	35	32	34	47	80	96
2	17	15	13	18	51	76	99
1	10	15	18	16	56	72	99
0.5	2	4	11	15	52	68	95
0	0	15	12	19	53	71	97
	0	1	2	4	8	16	32
				DPA 154			

A22							
64	92	93	94	93	92	93	92
32	83	92	93	90	94	93	94
16	78	73	77	78	75	76	93
8	57	52	48	51	72	77	87
4	42	39	43	45	41	38	46
2	29	28	31	32	35	31	46
1	8	3	15	24	32	33	42
0.5	4	5	12	18	21	37	38
0	0	2	17	22	28	34	45
	0	1	2	4	8	16	32
				DPA 156			

A22							
64	98	98	97	98	98	97	97
32	88	90	87	92	97	99	94
16	76	72	78	74	74	88	93
8	61	63	65	56	63	69	72
4	45	38	42	40	38	44	43
2	25	23	28	22	34	41	45
1	6	-1	7	5	18	26	47
0.5	5	2	2	11	15	18	43
0	0	5	8	12	17	21	42
	0	1	2	4	8	16	32
				H33258			

Figure S22. Synergistic action of A22 against *Escherichia coli* 25922. Checkerboard broth microdilution assays between A22 and top to bottom: DPA 154, DPA 156 and H33258. Dark regions represent higher cell density and numbers are the percent growth inhibition as

determined by optical density at A_{600nm}. Significant synergy is observed with the A22 / DPA 154 combination and no synergy with DPA 156 and H33258.

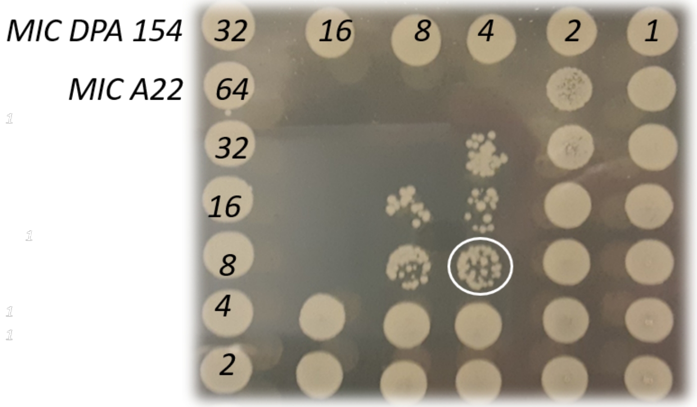

Figure S23. Representative example of spot plating from checkerboard synergy plate. *E. coli* 25922 was incubated with the A22/DPA154 combination 37°C for 16 h followed by spot plating 10 µL from each well onto tryptic soy agar plates and incubated at 37°C for 16 h for colony forming unit enumeration.

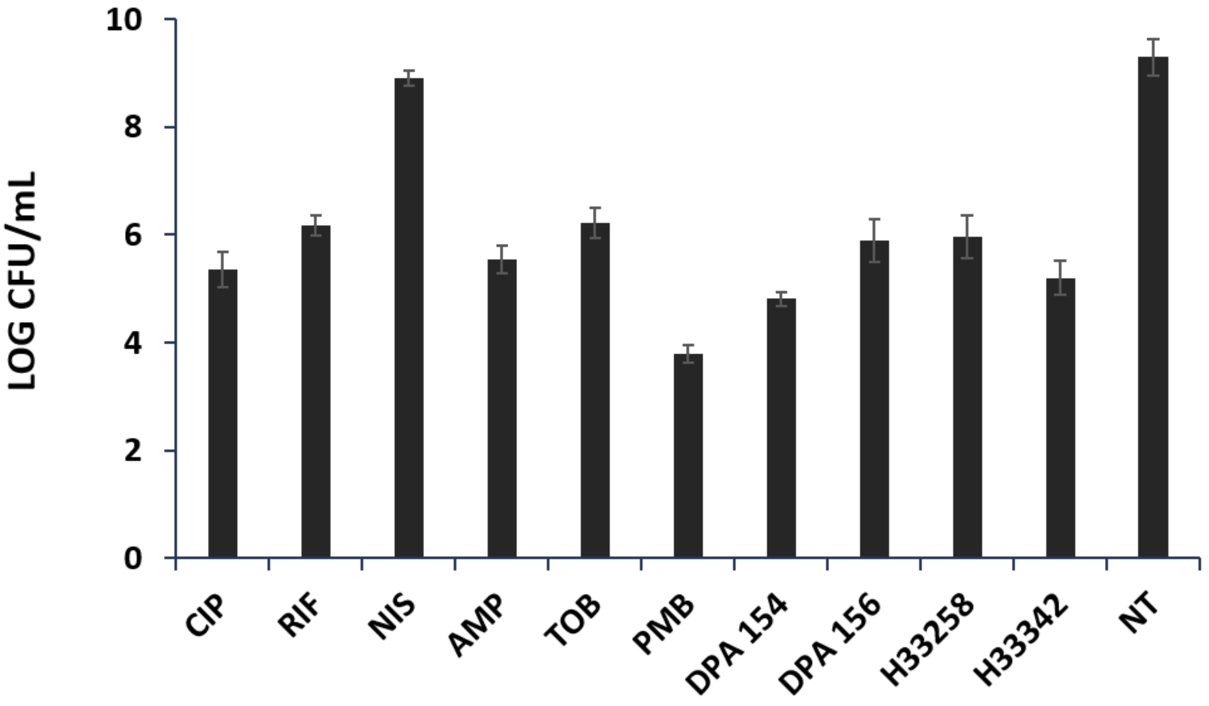

Figure S24 (addendum to SYTOX Green fluorescence experiment) Culturable number of *E. coli* cells treated with 5x MIC DPA 154, DPA 156 and antibiotics for 3 h. Abbreviations: CIP

ciprofloxacin, NIS nisin, RIF rifampicin, AMP ampicillin, TOB tobramycin, PMB polymyxin-B, H33258, H3342 Hoechst dye controls, NT no treatment. Error bars represent the standard deviation from the mean for assays performed on three occasions in duplicate.

Table S18. Minimal inhibitory concentration (MIC) for antibiotics in *E. coli* 25922.

Antibiotic	MIC (µM)
Ciprofloxacin	0.063 – 0.125
Rifampicin	2
Nisin	>50*
Ampicillin	4
Polymyxin B	0.625 – 1.25
DPA 154	12.5 – 25
DPA 156	25 – 50
Hoechst 33258	12.5 – 25
Hoechst 33342	6.25

MICs were performed in accordance to the Clinical Institute of Laboratory Standards using the broth microdilution method. * IC_{50}.

References

1. Klainer, A. S. & Perkins, R. L. Surface Manifestations of Antibiotic-Induced Alterations in Protein Synthesis in Bacterial Cells. *Antimicrob. Agents Chemother.* **1**, 164-170 (1972).

2. Cushnie, T. P. T., O'Driscoll, N.,H. & Lamb, A. J. Morphological and ultrastructural changes in bacterial cells as an indicator of antibacterial mechanism of action. *Cellular and Molecular Life Sciences* **73**, 4471-4492 (2016).

3. Higgins, M. L., Daneo-Moore, L., Boothby, D. & Shockman, G. D. Effect of inhibition of deoxyribonucleic acid and protein synthesis on the direction of cell wall growth in Streptococcus faecalis. *J. Bacteriol.* **118**, 681-692 (1974).

4. Spratt, B. G. & Pardee, A. B. Penicillin-binding proteins and cell shape in E. coli. *Nature* **254**, 516-516 (1975).

5. Henrich, B., Lubitz, W. & Plapp, R. Lysis of Escherichia coli by induction of cloned phi X174 genes. *Mol. Gen. Genet.* **185**, 493-497 (1982).

6. Diver, J. M. & Wise, R. Morphological and biochemical changes in Escherichia coli after exposure to ciprofloxacin. *J. Antimicrob. Chemother.* **18**, 31-41 (1986).

7. Elliott, T. S., Shelton, A. & Greenwood, D. The response of Escherichia coli to ciprofloxacin and norfloxacin. *J. Med. Microbiol.* **23**, 83-88 (1987).

8. Someya, A., Tanaka, K. & Tanaka, N. Morphological changes of Escherichia coli induced by bicyclomycin. *Antimicrob. Agents Chemother.* **16**, 87-91 (1979).

9. Schindler, P. R. & Teuber, M. Action of polymyxin B on bacterial membranes: morphological changes in the cytoplasm and in the outer membrane of Salmonella typhimurium and Escherichia coli B. *Antimicrob. Agents Chemother.* **8**, 95-104 (1975).

10. Kohanski, M. A., Dwyer, D. J. & Collins, J. J. How antibiotics kill bacteria: from targets to networks. *Nature reviews.Microbiology* **8**, 423-435 (2010).

11. Galizzi, A., Cacco, G., Siccardi, A. G. & Mazza, G. Mode of Action of Polymyxin B: Physiological Studies with a Bacillus subtilis-Resistant Mutant. *Antimicrob. Agents Chemother.* **8**, 366-369 (1975).

12. Wiedemann, I. *et al*. Specific binding of nisin to the peptidoglycan precursor lipid II combines pore formation and inhibition of cell wall biosynthesis for potent antibiotic activity. *J. Biol. Chem.* **276**, 1772-1779 (2001).

13. Nonejuie, P., Burkart, M., Pogliano, K. & Pogliano, J. Bacterial cytological profiling rapidly identifies the cellular pathways targeted by antibacterial molecules. *PNAS* **110**, 16169-16174 (2013).

14. Dewar, S., Reed, L. C. & Koerner, R. J. Emerging clinical role of pivmecillinam in the treatment of urinary tract infection in the context of multidrug-resistant bacteria. *J. Antimicrob. Chemother.* **69**, 303-308 (2014).

15. Bean, G. J. *et al*. A22 disrupts the bacterial actin cytoskeleton by directly binding and inducing a low-affinity state in MreB. *Biochemistry* **48**, 4852-4857 (2009).

16. Saleh, A. M. *et al*. Synthesis and biological evaluation of new pyridone-annelated isoindigos as anti-proliferative agents. *Molecules* **19**, 13076-13092 (2014).

17. Takacs, D. *et al*. Evaluation of forty new phenothiazine derivatives for activity against intrinsic efflux pump systems of reference Escherichia coli, Salmonella Enteritidis, Enterococcus faecalis and Staphylococcus aureus strains. *In Vivo* **25**, 719-724 (2011).

18. Ribera, A., Ruiz, J., Jiminez de Anta, M. T. & Vila, J. Effect of an efflux pump inhibitor on the MIC of nalidixic acid for Acinetobacter baumannii and Stenotrophomonas maltophilia clinical isolates. *J. Antimicrob. Chemother.* **49**, 697-698 (2002).

19. Klancnik, A. *et al*. Anti-Campylobacter activity of resveratrol and an extract from waste Pinot noir grape skins and seeds, and resistance of Camp. jejuni planktonic and biofilm cells, mediated via the CmeABC efflux pump. *J. Appl. Microbiol.* **122**, 65-77 (2017).

20. Dzoyem, J. P. *et al*. Cytotoxicity, antioxidant and antibacterial activity of four compounds produced by an endophytic fungus Epicoccum nigrum associated with Entada abyssinica. *Revista Brasileira de Farmacognosia* **27**, 251-253 (2017).

21. Lamers, R. P., Cavallari, J. F. & Burrows, L. L. The efflux inhibitor phenylalanine-arginine beta-naphthylamide (PAbetaN) permeabilizes the outer membrane of gram-negative bacteria. *PLoS One* **8**, e60666 (2013).

22. Brogden, R. N., Carmine, A. A., Heel, R. C., Speight, T. M. & Avery, G. S. Trimethoprim: a review of its antibacterial activity, pharmacokinetics and therapeutic use in urinary tract infections. *Drugs* **23**, 405-430 (1982).

23. Miller, M. J. Syntheses and therapeutic potential of hydroxamic acid based siderophores and analogs. *Chem. Rev.* **89**, 1563-1579 (1989).

24. Zavascki, A. P., Goldani, L. Z., Li, J. & Nation, R. L. Polymyxin B for the treatment of multidrug-resistant pathogens: A critical review. *J. Antimicrob. Chemother.* **60**, 1206-1215 (2007).

25. Velkov, T., Thompson, P. E., Nation, R. L. & Li, J. Structure--activity relationships of polymyxin antibiotics. *J. Med. Chem.* **53**, 1898-1916 (2010).

26. Thangamani, S. *et al*. Antibacterial activity and mechanism of action of auranofin against multi-drug resistant bacterial pathogens. *Sci. Rep.* **6**, 22571 (2016).

27. Ranjan, N. *et al*. Selective Inhibition of E. coli RNA and DNA Topoisomerase I by Hoechst 33258 Derived Mono and Bisbenzimidazoles. *J. Med. Chem.* **60 (12)**, 4904-4922 (2017).
